# Comparative Effects of Three Active Laser Therapy Protocols and a Sham Protocol on Neurophysiological and Functional Outcomes in Patients with Bell’s Palsy: A Four-Arm Randomized Controlled Trial

**DOI:** 10.3390/jcm15208113

**Published:** 2026-10-19

**Authors:** Ahmed Eid Lafi Aljohani, Mahmoud Ewidea, Amr Abo Gazia, Yasmein Mohammad

**Affiliations:** 1 Madina Cardiac Center, Madina 41311, Saudi Arabia; 2 Basic Sciences Department, Faculty of Physical Therapy, Kafr El Sheikh University, Kafr El Sheikh 33511, Egypt; mahewidea@gmail.com (M.E.); amr.abogazia@pua.edu.eg (A.A.G.); yasmeinyossef@gmail.com (Y.M.)

**Keywords:** Bell’s palsy, facial nerve palsy, high-intensity laser therapy, photobiomodulation, Nd:YAG laser, nerve conduction studies, facial disability index

## Abstract

**Background/Objectives:** Although laser therapy is commonly used to manage Bell’s palsy, optimal treatment parameters have not been established. The present randomized controlled trial compared three active laser therapy protocols with a sham-control protocol for neurophysiological and functional recovery in patients with acute Bell’s palsy. **Methods:** The primary analysis included all 104 randomized participants (26 per group) and used multiple imputation for missing six-week outcomes, followed by baseline-adjusted pooled treatment comparisons. In this four-arm trial (NCT07519421), 104 patients were randomized (26 per group) to receive either a sham laser, 808 nm low-level laser therapy (LLLT), 980 nm LLLT, or 1064 nm high-intensity laser therapy (HILT). All participants received standard care, including corticosteroids, electrical stimulation, and facial exercises. Interventions were administered three times per week for six weeks. Compound muscle action potential (CMAP) amplitude was the primary outcome; distal motor latency and the physical and social Facial Disability Index subscales (PFDI/SFDI) were additional outcomes. Eighty patients (20 per group) completed the six-week assessment and constituted the supporting complete-case analysis population. **Results:** In the primary intention-to-treat analysis, overall between-group differences were significant for CMAP amplitude (Wald χ^2^(3) = 1844.49), distal motor latency (χ^2^(3) = 284.90), PFDI (χ^2^(3) = 359.44), and SFDI (χ^2^(3) = 842.37; all *p* < 0.001). The Group C complete treatment protocol was superior to the sham, Group A, and Group B protocols on all four six-week outcomes in Bonferroni-adjusted pairwise comparisons (all *p* < 0.001). The 80-participant complete-case analysis supported the general direction of these findings. An additional baseline-adjusted complete-case sensitivity analysis showed broadly concordant treatment estimates and pairwise inference. No adverse events were reported. **Conclusions:** Among the complete treatment protocols evaluated, the 1064 nm HILT protocol was associated with the greatest short-term improvements at six weeks. Because the active protocols differed in multiple treatment parameters beyond wavelength, these findings should not be interpreted as demonstrating superiority of the 1064 nm wavelength itself. Longer-term multicenter trials are needed to confirm durability and generalizability. The ITT estimates rely on a missing-at-random assumption for the 24 participants without six-week outcome assessments. Post-imputation diagnostic checks identified departures from regression assumptions, particularly for functional outcomes; inferential results should therefore be interpreted cautiously.

## 1. Introduction

Facial paralysis is an acute neurological disorder characterized by dysfunction of the seventh cranial nerve, typically affecting one side of the face. Affected individuals experience unilateral or, less commonly, bilateral facial weakness and drooping. Associated symptoms may include fever, hyperacusis, postauricular pain, and ocular dryness. Viral infections and ischemia are the primary etiologies of facial paralysis [1]. Fifty to 60% of cases of peripheral facial palsy are Bell’s palsy, with an annual incidence of 8–52.8 per 100,000 people; between 69% and 85% of patients recover spontaneously. Incomplete recovery accounts for 30% of cases and is accompanied by some functional and psychological issues [2].

The prognosis differs substantially according to the extent of nerve damage and the timing of intervention. Steroid therapy is widely recognized as the first-line treatment for Bell’s palsy because of its role in accelerating recovery. For optimal outcomes, steroid therapy is recommended within 72 h of symptom onset. The addition of antiviral medications may be considered in severe cases, as suggested by recent guidelines, although the efficacy of this combination remains under investigation [3].

Some studies have reported that combined therapy does not improve symptom resolution in patients with Bell’s palsy [4], whereas others have demonstrated its effectiveness in reducing long-term sequelae [5].

Electroneurography (ENoG) assesses the evoked compound muscle action potential (CMAP) of targeted facial muscles following transcutaneous stimulation of the facial nerve trunk. ENoG findings may remain normal during the first three days after injury and typically stabilize by approximately 21 days; therefore, the interval from 3 to 21 days is considered the principal prognostic window [6]. More recent work has described a “time-continuous approach” in which serial ENoG measurements are used to monitor recovery dynamically over time [7].

After one year, 71% of patients with facial nerve paralysis achieved complete spontaneous recovery without treatment, while 13% experienced mild weakness, and 16% demonstrated moderate to poor recovery. Consequently, considerable debate persists regarding the most appropriate treatment for this condition [8]. The Sunnybrook facial grading system (SFGS) and the facial disability index (FDI) are commonly used to quantify the effects of facial exercises on early recovery, muscle strength, and symmetry in patients with Bell’s palsy [9].

Despite the use of steroids and facial expression exercises, only 30% of patients with Bell’s palsy achieve complete recovery [10]. While several studies have demonstrated the effectiveness of electrical stimulation in promoting recovery, its impact and efficacy in acute cases remain under investigation [11]. Current evidence indicates that electrical stimulation does not confer additional benefit when combined with pharmacological or nonpharmacological treatments in patients with acute Bell’s palsy [12].

Accumulating clinical evidence supports laser therapy as a potential adjunctive treatment for Bell’s palsy. Previous studies have reported improvements in disease severity, facial symmetry, and facial function following laser therapy [13]. Photobiomodulation has also shown favorable responses in acute and subacute Bell’s palsy, with greater improvement reported than in chronic cases [14].

When laser therapy is used therapeutically, appropriate selection of treatment parameters is essential because these parameters influence tissue penetration and the biological effects achieved at the target depth. Wavelength is particularly important because it substantially affects penetration depth, reflecting the distinct absorption spectra of tissue chromophores [15]. The protocols evaluated in this study were selected on the premise that greater light penetration may facilitate delivery of energy to deeper facial nerve structures [16,17].

The active protocols also have distinct proposed photophysical and cellular mechanisms. At approximately 808 nm, photobiomodulation has been investigated in relation to mitochondrial electron-transport activity and cytochrome-c oxidase-associated signaling, although the precise initiating chromophores and responses remain context dependent [18,19]. At 980 nm, interactions with tissue water and other absorbers may contribute to a different balance of photobiomodulatory and thermal effects [17]. The 1064 nm Nd:YAG approach has been discussed in relation to tissue optical properties, energy deposition, and photothermal responses, which depend not only on wavelength but also on power, exposure conditions, and tissue composition [20,21]. These mechanistic distinctions informed the rationale for comparing the complete protocols; they were not directly measured or experimentally separated in this trial.

To date, there has been no direct comparison of three active laser therapy protocols and a sham-control protocol on ENoG latency and amplitude outcomes in patients with acute Bell’s palsy. The active protocols evaluated in this study differed in wavelength, total energy output, treatment area, power, application sites, and delivery characteristics; therefore, they were evaluated as complete intervention protocols rather than as isolated wavelength-specific interventions. Given the heterogeneity of previous phototherapy studies, well-designed and standardized randomized clinical trials of laser treatment protocols for Bell’s palsy are needed [22].

Therefore, the present study aimed to compare three active laser therapy protocols (808 nm LLLT, 980 nm LLLT, and 1064 nm HILT) with a sham-control protocol on neurophysiological and functional outcomes in patients with acute Bell’s palsy. CMAP amplitude was specified as the primary outcome, while distal motor latency, PFDI, and SFDI were evaluated as additional outcomes. We hypothesized that the active laser therapy protocols would produce greater short-term neurophysiological and functional improvements than the sham-control protocol and that the magnitude of improvement would differ among the active protocols.

## 2. Materials and Methods

### 2.1. Study Design and Participants

Patients with Bell’s palsy were recruited from King Fahd Hospital in Al-Madinah, Saudi Arabia. Ethical approval for the study was obtained from the institutional review board of Madinah Cardiac Center, Saudi Arabia (No. MCC-143-2513) and was registered retrospectively at ClinicalTrials.gov (NCT07519421). However, all mandatory institutional ethical approvals were fully secured before the initiation of the study, and the trial was conducted strictly according to the pre-approved protocol. Additionally, academic ethical clearance was obtained from the Scientific Research Ethics Committee of Kafr El-Sheikh University, Egypt (FSIRB200-675, 29 June 2025), for postgraduate research oversight. The final treatment session for the last enrolled participant was completed on 27 May 2026. Written informed consent was obtained from all participants following the provision of relevant information regarding the study’s purpose, procedures, potential benefits, and confidentiality. This four-arm study included male and female patients aged 18–70 years with unilateral facial involvement. Assessment was conducted via the House–Brackmann grading scale (HBGS), with inclusion criteria of moderate to severe (grade III or higher) [23]. All patients received standard treatment for acute Bell’s palsy, consisting of oral steroids and antiviral medications, within 72 h of symptom onset [3]. The exclusion criteria included central nervous system pathology and recurrent Bell’s palsy [24]. Age under 18 years, mental illness or social issues, bilateral facial palsy, malignancy, temporomandibular joint (TMJ) manifestations, facial dysmorphism, pregnancy planning, pregnancy, or lactation.

Trial registration and timing. The trial was retrospectively registered on ClinicalTrials.gov (NCT07519421). The registration was first submitted on 1 April 2026 and first publicly posted on 9 April 2026, after participant enrollment had commenced. This delay resulted from an unintentional administrative oversight concerning the requirement to register a randomized clinical trial before enrolling the first participant. Although institutional ethical approvals had been obtained before enrollment, the research team initially misunderstood the timing requirement for public registration. Upon recognizing the requirement, the registration process was initiated. Ethical approval does not replace prospective trial registration.

No important changes were made to the trial design, eligibility criteria, or intervention procedures after trial commencement.

### 2.2. Sample Size Calculation

An a priori sample-size calculation was performed using G*Power (version 3.1.9.7) for the primary outcome, CMAP amplitude, using a four-group × two-time-point mixed repeated-measures ANOVA framework. Assuming a medium effect size (Cohen’s f = 0.25), α = 0.05, and 95% statistical power, a minimum of 76 evaluable participants (19 per arm) was required. To account for an anticipated attrition rate of approximately 15%, the recruitment target was increased to 92 participants (23 per arm), and an additional allowance for attrition resulted in 104 participants being randomized (26 per arm). Although 24 participants did not provide the six-week outcome assessment, 80 participants remained evaluable (20 per arm). Thus, the final evaluable sample remained above the prespecified minimum requirement of 76 participants (19 per arm) and satisfied the original a priori sample-size requirement under the assumptions used in the power calculation.

### 2.3. Randomization, Allocation Concealment, and Blinding

An independent biostatistician generated the random allocation sequence usingMicrosoft Excel (version 16.61.1 [22052000]; RAND function)with permuted blocks of four or eight (1:1:1:1 ratio). Clinical staff in the outpatient department screened potential participants and enrolled those who were eligible. To maintain allocation concealment, sequentially numbered, opaque, sealed foil envelopes were used. Following baseline examinations, an independent research assistant—who had no further role in patient management or outcome assessment—opened the sequential envelopes and assigned each participant to their designated intervention. Participants were intended to remain blinded to active versus sham allocation, and outcome assessors were blinded throughout the trial, whereas treating physical therapists were not. All participants wore opaque protective goggles during treatment, and integrated cooling was used across all treatment arms to promote uniform thermal perception. The sham device delivered zero optical output (0 mW; 0 J/cm^2^) while reproducing visual and auditory device cues. These measures were implemented to support participant blinding; however, blinding effectiveness was not formally assessed by asking participants to guess their allocation. The study design and reporting followed the Consolidated Standards of Reporting Trials (CONSORT) guidelines [25]. Figure 1 presents the participant flow through enrollment, allocation, follow-up, and analysis.

CONSORT analysis-population note: All 104 randomized participants (26 per arm) were retained in the primary intention-to-treat analysis; 80 completed the six-week assessment (20 per arm), and 24 had missing six-week outcomes (six per arm). The 80-completer analyses are supportive complete-case analyses.

**Primary outcome measurement**
**Electroneurography (ENoG)**

Electroneurography (ENoG) was performed using the Dantec™ Keypoint^®^ Focus 3-Channel EMG/NCS/EP System (Natus Manufacturing Limited, Gort, County Galway, Ireland). Electrical stimulation was administered to the main trunk of the facial nerve supramaximally at its exit from the stylomastoid foramen via a bipolar stimulator or stimulating electrodes. A bipolar configuration of surface electrodes placed on the target muscle recorded the compound muscle action potential (CMAP). The standard method was employed, with ENoG recorded at the nasolabial fold, which is recognized as the most reliable recording site [26] (Figure 2). Measurements were first performed on the healthy side of the face, followed by the affected side. All patients were assessed at baseline and six weeks after treatment.

**Prearrangements**

To reduce retest variability, room temperature was maintained at a constant level, equipment was regularly inspected, electrical shielding was ensured, and the skin where the electrodes and stimulator were applied was cleaned with alcohol. Participants were positioned in an adjustable chair or on an examination couch.

**Parameters**

The recording parameters included a sensitivity setting of 10,000 mV, amplifier filtering between 1 and 10 kHz, and a time base of 10 ms. The stimulus duration was 200 μs, with a maximal stimulus limit of 20 mA and a stimulus rate of 1.9 s. Data were recorded and averaged at a stimulus rate of 1 Hz, with sensitivity set to 2 μV per division and filters set to 30 Hz–3 kHz. Ground electrodes were placed on the arm or neck. Stimulation was first applied to the healthy side, followed by the paralyzed side. Recording electrodes were positioned on the adjacent nasal alae, and stimulation was delivered at the stylomastoid groove, starting at 0.1 mA and increasing until the maximum compound muscle action potential (CMAP) was achieved. Stimulation was then slightly increased to achieve supramaximal stimulation. The CMAP was stored, and the procedure was repeated on the contralateral side [6].

**Secondary outcome measurement**
**Evaluation of Facial Recovery**

The Facial Disability Index (FDI) is a commonly used patient-reported measure for assessing disability associated with Bell’s palsy. It was designed primarily to evaluate both the physical and social aspects of facial function. The index has a total score of 100 points [27]. Each component is scored separately: one physical subscale (PFDI) and another social subscale (SFDI). Higher scores indicate greater improvement in facial function. Patients underwent assessment at baseline and again at six weeks [24].

**Interventions**
**Standard Protocol for All Groups**

**a-**  **Medical management (corticosteroids and antiviral course)**

All participants received treatment in accordance with the National Institutes of Health (NIH) guidelines for acute Bell’s palsy, as recommended in previous studies [11,12]. The medical regimen included prednisone at a dose of 60 mg daily for the first 5 days, followed by a gradual reduction to a 10 mg dose per day over the next 5 days. In addition, participants were administered oral acyclovir or valacyclovir for the initial 5 days, which is consistent with established protocols [3].

**Mandatory Eye Care Protocol**

The American Academy of Otolaryngology-Head and Neck Surgery recommends immediate and aggressive eye protection for patients with impaired eye closure to prevent permanent corneal damage. Daytime measures include lubricating eye drops every 1–2 h while awake and wearing sunglasses outdoors to protect against wind and particles. Nighttime measures include ophthalmic ointment at bedtime for sustained moisture and eye taping or patching with careful instructions on proper techniques to avoid corneal abrasion [28].

**b-**  **Electrical stimulation**

Adjunctive electrical stimulation (ES) therapy during acute Bell’s palsy may reduce the risk of incomplete recovery [11]. ES was administered to all participants via an interrupted galvanic current, delivering 100 millisecond rectangular pulses at a rate of one pulse per second to each facial muscle. This protocol evoked three sets of 30 minimal contractions per day in a single session. ES was provided daily (six days per week) until active contractions returned, after which it was administered on alternate days, for a total duration of three weeks from the initial patient visit (within seven days of onset) [12].

**c-**  **Standard facial exercise protocol**

Following electrical stimulation, participants performed supervised mimetic facial exercises in front of a mirror in accordance with NHS facial rehabilitation guidance. The regimen incorporated five repetitions of targeted movements—including brow elevation, gentle frowning, guided eye closure, symmetric cheek puffing, and smile variations—performed 4–5 times daily, five days per week over the six-week intervention period [29].

**Laser Therapy Protocols**
**Interventions**
**Sham-Control Group**

The sham-control intervention used an inactive device providing zero optical output (0 mW; 0 J/cm^2^) and therefore delivered no therapeutic laser energy. The procedure reproduced the procedural characteristics of active treatment as closely as feasible, including visual indicators, operational audio tones, treatment contact, and contact duration across eight anatomical landmarks (60 s per point; 480 s per session). Eighteen sessions were conducted over six consecutive weeks (three sessions per week) [30]. All participants wore opaque protective goggles, and integrated cooling was used across treatment arms to minimize differences in perceived thermal sensation. These procedures were intended to minimize participants’ ability to distinguish sham from active treatment; however, the effectiveness of participant blinding was not formally assessed. The sham methodology was consistent with established protocols reported in previous multiarm laser trials [31].

**Study Group (A)**

Laser therapy was administered using the B-Cure Laser Pro device (Good Energies Ltd., Haifa, Israel), a class II laser according to the ANSI classification. Device calibration adhered strictly to manufacturer standards: the B-Cure Laser Pro (808 nm) was fully recharged prior to each session with factory-preset dosimetry. The device emits in the near-infrared spectrum (808 nm) and features a green LED aiming beam to indicate the irradiated area of 4.5 cm^2^. Its output power of 250 mW is delivered in micropulses at a frequency of 15 kHz, providing an energy per minute of 14.4 J and a fluence per minute of 3.2 J/cm^2^. Treatment was performed by cutaneous application for 15 min per session (total fluence: 48 J/cm^2^), with the device placed in contact with the skin over the right parotid gland, as described in previous case reports [32]. Patients received 18 sessions of laser therapy three times per week for six consecutive weeks [33].

**Study Group (B)**

Laser therapy was delivered using a low-level laser device (model BTL-4000; BTL Industries Ltd., Stevenage, UK). The diode emitted at a wavelength of 980 nm, with a frequency of 100 Hz and an energy density of 5 J per point, was applied by direct contact with the facial skin at nine designated points for one minute each. Device calibration adhered strictly to manufacturer standards: The BTL-4000 device (980 nm) utilized integrated real-time self-calibration to verify beam intensity. The contact sites on the affected side included locations near the mastoid process at the stylomastoid foramen and two points along each pathway of the facial nerve branches, as determined by previous studies. These sites included the temporal branch in the forehead, the zygomatic nerve along the zygomatic arch, the buccal nerve in the mid-cheek over the buccal muscles, and the mandibular branches in the chin area [34]. Laser therapy was administered in 18 sessions over six consecutive weeks (three sessions per week) as shown in Figure 3.

**Study Group (C)**

This group received high-intensity laser therapy (HILT) using a pulsed Nd:YAG laser (HIRO 3 device; ASA S.r.l., Arcugnano, Vicenza, Italy). The device was operated with pulsed emission at 1064 nm, a peak power of 3 kW, an energy density of 810–1780 mJ/cm^2^, a pulse duration of 120–150 μs, a frequency of 10–40 Hz, and a duty cycle of approximately 0.1%. HILT was applied in contact with and perpendicular to the superficial roots of the facial nerve on the affected side. Each point received 7 s of application at an energy density of 10 J/cm^2^, with a total energy delivery of 80 joules per session. Device calibration adhered strictly to manufacturer standards: the HIRO 3 device (1064 nm) Nd:YAG laser underwent authorized factory servicing with automated internal energy monitoring. The device automatically calculates the number of pulses, energy per session, and total energy delivered. HILT was also administered in 18 sessions over six consecutive weeks (three sessions per week) [33].

**General precautions and adverse events**

The study adhered to the World Association of Laser Therapy (WALT) guidelines for low-level laser therapy (LLLT) intervention [35]. No formal interim analyses or stopping guidelines for trial futility/early efficacy were planned or conducted. Individual treatment cessation criteria were prespecified strictly for safety monitoring (skin temperature > 42 °C, intolerable pain, or unexpected erythema). To ensure safety during laser application, both investigators and participants wore protective goggles to prevent potential ocular damage, and all adverse events were recorded and monitored by the research investigator. Real-time skin temperature monitoring was conducted via a forward-looking infrared (FLIR) thermal imaging camera. Skin temperature was recorded at the treatment site before, during, and immediately after each session. This finding aligns with established protocols for thermal monitoring during laser therapy to maintain safe temperature ranges and prevent thermal injuries [36]. The stopping rules implemented in this study were consistent with the intraoperative safety precautions and complication management standards established in the international consensus statement on facial laser procedures, which emphasize careful patient monitoring and predefined criteria for intervention to prevent adverse events [37].

**Statistical analysis**

Primary analyses followed the intention-to-treat (ITT) principle and included all 104 randomized participants in their assigned groups. The primary estimand was the difference between randomized groups in six-week outcomes, adjusted for the corresponding baseline measurement. The primary ITT analysis was independently implemented in Python (version 3.12) using multiple imputation (MI), baseline-adjusted ordinary least-squares regression, and Rubin’s rules. Continuous variables are summarized as mean ± standard deviation and categorical variables as counts and percentages. The original complete-case analyses of the 80 participants with observed six-week outcomes were retained as supportive sensitivity analyses.

Missing six-week values of distal motor latency, CMAP amplitude, PFDI, and SFDI were imputed under the missing-at-random (MAR) assumption with stochastic Bayesian fully conditional regression (50 completed datasets; seed 20260928). Predictors in the reported specification included randomized group, age, onset-to-baseline interval, sex, House–Brackmann grade, diabetes, retroauricular pain, and baseline values of all four outcomes, with other imputed follow-up outcomes included in each conditional model. Only missing six-week outcomes were replaced; observed values were retained. Imputed PFDI/SFDI values were within 0–100 and CMAP amplitudes were nonnegative. All 50 saved completed datasets were independently audited against the observed raw data. Each dataset was analyzed using baseline-adjusted ordinary least squares; coefficients and covariance matrices were pooled by Rubin’s rules. Overall group effects were reported using an asymptotic pooled Wald χ^2^ approximation (3 df). Six pairwise contrasts per outcome used MI-adjusted degrees of freedom with Bonferroni-adjusted *p*-values and family-wise 95% confidence intervals. The 50 saved completed datasets and analysis materials supporting the pooled estimates were retained for verification. The missing-at-random assumption cannot be confirmed from the observed data.

Multiplicity and outcome hierarchy. CMAP amplitude was designated as the primary outcome. The primary omnibus treatment-group test was evaluated first; additional outcomes (distal motor latency, PFDI and SFDI) were interpreted as secondary, supportive evidence rather than as independently confirmatory co-primary endpoints. Within each outcome, the six pairwise treatment comparisons were adjusted using Bonferroni correction (family-wise alpha = 0.05; two-sided individual threshold 0.05/6), with correspondingly simultaneous 95% confidence intervals. This correction applies within, not across, the four outcome families; therefore, secondary-outcome results should be interpreted cautiously in light of multiplicity across outcomes. No post hoc change to the reported treatment-effect estimates or significance decisions was made on the basis of these diagnostics.

Post-imputation model diagnostics. Using the 50 retained completed datasets underlying the reported pooled ITT estimates, we assessed residual normality by Shapiro–Wilk tests and Q–Q plots, heteroscedasticity by Breusch–Pagan tests and residual-versus-fitted plots, and potential influence using Cook’s distance (4/n as a screening threshold, not an exclusion rule). Across the 50 models per outcome, Shapiro–Wilk tests were significant in 45/50 Distal motor latency, 30/50 CMAP amplitude, 40/50 PFDI, 8/50 SFDI; Breusch–Pagan tests were significant in 2/50 Distal motor latency, 1/50 CMAP amplitude, 33/50 PFDI, 32/50 SFDI. Median numbers of observations exceeding Cook’s D > 4/104 were 5 for Distal motor latency, 4 for CMAP amplitude, 6 for PFDI, 8 for SFDI. The fractions of missing information for treatment coefficients ranged from 0.179 to 0.307. Monte Carlo standard errors for the pooled treatment coefficients were calculated as sqrt(B/m), where B denotes between-imputation variance and m = 50. These diagnostics describe model assumptions and imputation uncertainty; they do not validate the MAR assumption or establish robustness to departures from it.Representative diagnostic figures are provided in Supplementary Figures S1–S12; descriptive complete-case figures are provided in Supplementary Figures S13–S20.

The original mixed-design MANOVA, outcome-specific mixed ANOVAs, and descriptive analyses in the 80 completers are presented as supportive complete-case analyses. Because these analyses use a different population and model specification, their test statistics are not directly interchangeable with the primary baseline-adjusted ITT estimates. Missing-at-random is untestable, and imputation does not eliminate potential bias due to unobserved causes of missingness.

The complete-case mixed MANOVA and outcome-specific mixed ANOVAs were originally planned as observed-data analyses and are now reported as supportive analyses. No exploratory subgroup analyses were undertaken.

## 3. Results

Results are presented in the following order: baseline characteristics of all 104 randomized participants, primary intention-to-treat (ITT) comparisons using 50 multiply imputed datasets, and supportive complete-case analyses of the 80 participants with observed six-week outcomes. The pooled baseline-adjusted ITT and complete-case mixed-model statistics address different analysis populations and should not be interpreted as interchangeable.

### 3.1. Baseline Characteristics of the Randomized Population (n = 104)

Baseline characteristics for all 104 randomized participants (26 per arm), including baseline neurophysiological and functional outcomes, are summarized in Table 1. Inferential baseline comparisons are reported in response to the reviewers’ request; these exploratory tests should not be interpreted as tests of the success of randomization.

#### 3.1.1. Sex Distribution

Sex distribution among all 104 randomized participants (26 per group) is presented in Table 2. The distribution is descriptive and is not restricted to the 80 six-week completers.

#### 3.1.2. Affected-Side Distribution

The distribution of the affected side among all 104 randomized participants (26 per group) is presented in Table 3.

### 3.2. Primary Intention-to-Treat Analysis (n = 104) and Multiple Imputation

Analysis populations and attrition. All 104 randomized participants (26 per arm) were retained in the primary ITT analysis. Six-week outcomes were observed for 80 participants (20 per arm); 24 participants had missing six-week outcomes (six per arm; 23.1% overall). Missing six-week values were multiply imputed, and the 80 completers were analyzed separately as a supporting complete-case analysis (Table 4).

Primary ITT and multiple imputation. Missing six-week distal motor latency, CMAP amplitude, PFDI, and SFDI values were imputed under a missing-at-random assumption using 50 completed datasets generated using stochastic Bayesian fully conditional regression (seed 20260928). The reported imputation specification included randomized group, age, onset-to-baseline interval, sex, House–Brackmann grade, diabetes, retroauricular pain, baseline values of all four outcomes, and other imputed follow-up outcomes. Observed follow-up values were retained in every completed dataset; imputed PFDI/SFDI values were within 0–100 and CMAP amplitudes were nonnegative. For this revision, all 50 saved completed datasets were independently audited against the original observed data and reanalyzed with baseline-adjusted outcome-specific linear models. Coefficients and covariance matrices were combined using Rubin’s rules. Six pairwise contrasts per outcome used Bonferroni-adjusted *p*-values and family-wise 95% confidence intervals. The complete datasets and reproducible pooling script are retained as analysis materials. The pooled results reported below were calculated from the 50 retained completed datasets.

Assessment of the missingness pattern showed that participants without six-week outcomes were older (44.00 vs. 36.53 years; *p* = 0.003), had a longer interval from symptom onset to baseline assessment (45.62 vs. 38.66 h; *p* = 0.030), and had slightly higher baseline affected-side latency (4.857 vs. 4.819 ms; *p* = 0.019) than completers. No statistically significant differences were detected for baseline CMAP amplitude, PFDI, SFDI, sex, House–Brackmann grade, diabetes, retroauricular pain, or randomized treatment arm. These observed predictors of missingness were therefore represented in the imputation model where applicable. The MAR assumption remains an unverifiable modeling assumption and residual bias from unmeasured causes of attrition cannot be excluded. Because the imputation model assumes missingness at random conditional on included variables, these comparisons should be interpreted alongside the missing-data limitations described below.

Primary ITT treatment effects. Pooled adjusted mean differences, Bonferroni family-wise 95% confidence intervals, and adjusted p-values for all six pairwise contrasts per outcome are presented in Table 5. Across 50 completed imputed datasets, the pooled baseline-adjusted group effects were significant for latency (approximate Wald χ2(3) = 284.90), CMAP amplitude (χ2(3) = 1844.49), PFDI (χ2(3) = 359.44), and SFDI (χ2(3) = 842.37; all asymptotic *p* < 0.001; Table 6). The Group C protocol was significantly more favorable than each of the other three complete protocols for all four six-week outcomes. Study Groups A and B were not significantly different for latency or CMAP amplitude. These are between-protocol comparisons and do not isolate wavelength effects.

Primary ITT model diagnostics. Across the 50 completed datasets, residual-normality tests indicated departures from normality in 30/50 CMAP models, 45/50 latency models, 40/50 PFDI models, and 8/50 SFDI models. Breusch–Pagan tests suggested heteroscedasticity in 1/50 CMAP models, 2/50 latency models, 33/50 PFDI models, and 32/50 SFDI models. Median counts of observations exceeding the Cook’s distance screening threshold (4/104) were 4, 5, 6, and 8 for CMAP, latency, PFDI, and SFDI, respectively. These descriptive diagnostic frequencies indicate that model assumptions, particularly those for the functional outcomes, warrant caution; they are not additional tests of treatment efficacy. No observations were excluded on the basis of Cook’s distance.

The supporting complete-case analyses showed broadly concordant directions of effects but were based on a different analysis population and model specification. The MAR assumption is not empirically verifiable, and the ITT analysis cannot exclude bias if missingness depends on unobserved six-week outcomes. The pooled estimates were checked against the 50 retained completed datasets. These checks support the reported pooled calculations but do not, by themselves, establish that the missing-at-random assumption holds.

The trial randomized 104 participants (26 per arm); 80 had observed six-week assessments (20 per arm) and 24 lacked the six-week outcomes (6 per arm; 23.1% attrition). The primary ITT analysis retained all 104 randomized participants through multiple imputation of missing follow-up outcomes. The 80-completer analyses were used as supporting sensitivity analyses.

Post-randomization attrition was evenly distributed across the four trial arms, with six participants lost from each arm. Importantly, none of these withdrawals were related to adverse events, safety concerns, or intolerance to the assigned interventions. Rather, attrition was driven entirely by personal and logistical challenges. Within the sham-control group, dropouts occurred due to scheduling conflicts (*n* = 3), transportation issues (*n* = 2), and a single withdrawal of consent. The active treatment arms experienced similar practical barriers: Study Group A losses stemmed from scheduling conflicts (*n* = 3), geographic relocation (*n* = 2), and loss to follow-up (*n* = 1); Study Group B attrition was due to scheduling constraints (*n* = 4) and loss to follow-up (*n* = 2); and Study Group C participants withdrew because of scheduling conflicts (*n* = 3), transportation barriers (*n* = 2), and relocation (*n* = 1).

#### Sensitivity Analysis: ITT Versus Complete Cases (Baseline-Adjusted Models)

To assess sensitivity to the analysis population, the original 80 observed six-week cases (20 per group) were reanalyzed using the same outcome-specific baseline-adjusted linear-model structure as the primary multiple-imputation ITT analysis (104 randomized participants; 50 imputations). Six treatment contrasts per outcome were tested with Bonferroni-adjusted two-sided *p*-values; complete-case family-wise 95% confidence intervals used the corresponding t critical value. The ITT estimates in the comparison below are those already reported in Table 5, not newly generated imputations.

In Table 7, MD = adjusted mean difference (first minus second); FW CI = family-wise confidence interval; CC = complete case. Adjustment: corresponding baseline outcome. The CC − ITT column is a descriptive difference between point estimates, not a hypothesis test.

The overall complete-case group effect remained significant for CMAP amplitude (*p* < 0.001), latency (*p* < 0.001), PFDI (*p* < 0.001), and SFDI (*p* < 0.001). Bonferroni-adjusted significance classifications agreed for 24 of 24 ITT-versus-complete-case pairwise contrasts. The largest clinical ordering pattern—more favorable six-week outcomes for Group C than the other arms—was preserved across the four outcomes; the comparison nevertheless evaluates sensitivity to excluding missing outcomes and does not establish robustness to missing-not-at-random departures.

### 3.3. Supporting Complete-Case Analysis (n = 80): Mixed MANOVA and Outcome-Specific Mixed ANOVAs

For the supporting complete-case analysis, improved distal motor latency is indicated by a decrease in milliseconds, whereas improved CMAP amplitude is indicated by an increase in millivolts. Table 8 presents the statistical assumption checks and mixed-design MANOVA results. Table 9, Table 10, Table 11 and Table 12 summarize the original observed-data outcome-specific results for the 80 completers.

In the supporting complete-case analysis (*n* = 80), mixed-design MANOVA used Pillai’s trace. The original complete-case tests showed significant group, time, and group × time effects (all *p* < 0.001; Table 8). These results are supportive and are not the primary ITT treatment estimates.

#### 3.3.1. Effect of Treatment on Latency

Within-group comparisons. Distal motor latency decreased significantly in all four groups. In the control group, mean ± SD latency decreased from 4.82 ± 0.08 ms to 4.11 ± 0.08 ms (mean change −0.71 ms; 15% reduction; 95% CI: −0.764 to −0.651; *p* < 0.001). Study Group A decreased from 4.82 ± 0.08 ms to 3.82 ± 0.12 ms (mean change −1.00 ms; 21% reduction; 95% CI: −1.054 to −0.941; *p* < 0.001), Study Group B from 4.82 ± 0.08 ms to 3.85 ± 0.06 ms (mean change −0.97 ms; 20% reduction; 95% CI: −1.029 to −0.916; *p* < 0.001), and Study Group C from 4.83 ± 0.07 ms to 3.63 ± 0.09 ms (mean change −1.20 ms; 25% reduction; 95% CI: −1.251 to −1.139; *p* < 0.001) (Table 9).

Between-group comparisons. No significant baseline difference in latency was detected among the four groups (*p* = 0.986). At six weeks, latency was significantly greater in the control group (4.11 ± 0.08 ms) than in Study Groups A (3.82 ± 0.12 ms), B (3.85 ± 0.06 ms), and C (3.63 ± 0.09 ms) (all *p* < 0.001). Groups A and B did not differ significantly (*p* = 1.000), whereas Group C had significantly lower latency than both Groups A and B (both *p* < 0.001) (Table 9).

#### 3.3.2. Effect of Treatment on CMAP Amplitude

Within-group comparisons. CMAP amplitude increased significantly in all four groups. In the control group, mean ± SD amplitude increased from 0.65 ± 0.05 mV to 1.18 ± 0.11 mV (mean change 0.53 mV; 82% increase; 95% CI: 0.478 to 0.582; *p* < 0.001). Study Group A increased from 0.64 ± 0.06 mV to 2.10 ± 0.08 mV (mean change 1.46 mV; 228% increase; 95% CI: 1.403 to 1.507; *p* < 0.001), Study Group B from 0.64 ± 0.06 mV to 2.06 ± 0.09 mV (mean change 1.42 mV; 222% increase; 95% CI: 1.373 to 1.477; *p* < 0.001), and Study Group C from 0.62 ± 0.07 mV to 2.61 ± 0.11 mV (mean change 1.99 mV; 321% increase; 95% CI: 1.943 to 2.047; *p* < 0.001) (Table 10).

Between-group comparisons. No significant baseline difference in CMAP amplitude was detected among the four groups (*p* = 0.439). At six weeks, amplitude was significantly lower in the control group (1.18 ± 0.11 mV) than in Study Groups A (2.10 ± 0.08 mV), B (2.06 ± 0.09 mV), and C (2.61 ± 0.11 mV) (all *p* < 0.001). Groups A and B did not differ significantly (*p* = 1.000), whereas Group C had significantly greater amplitude than both Groups A and B (both *p* < 0.001) (Table 10).

#### 3.3.3. Effect of Treatment on PFDI

Within-group comparisons. The control-group PFDI changed from 24.75 ± 11.06% to 25.75 ± 10.92% (mean change 1.00 percentage point; 4% increase; 95% CI: −3.352 to 5.352; *p* = 0.649). Study Group A increased from 22.25 ± 13.13% to 33.50 ± 17.18% (mean change 11.25 percentage points; 51% increase; 95% CI: 6.898 to 15.602; *p* < 0.001), Group B from 28.00 ± 10.05% to 59.75 ± 16.58% (mean change 31.75 percentage points; 113% increase; 95% CI: 27.398 to 36.102; *p* < 0.001), and Group C from 20.75 ± 8.63% to 77.75 ± 11.86% (mean change 57.00 percentage points; 275% increase; 95% CI: 52.648 to 61.352; *p* < 0.001) (Table 11).

Between-group comparisons. No significant baseline difference in PFDI was detected among the four groups (*p* = 0.172). At six weeks, the control group (25.75 ± 10.92%) did not differ significantly from Group A (33.50 ± 17.18%; *p* = 0.558), but had significantly lower scores than Groups B (59.75 ± 16.58%; *p* < 0.001) and C (77.75 ± 11.86%; *p* < 0.001). Group A had significantly lower scores than Groups B and C, and Group B had significantly lower scores than Group C (all *p* ≤ 0.001) (Table 11).

#### 3.3.4. Effect of Treatment on SFDI

Within-group comparisons. The control-group SFDI changed from 24.20 ± 8.94% to 25.00 ± 8.89% (mean change 0.80 percentage points; 95% CI: −2.11 to 3.71; *p* = 0.586). Study Group A increased from 24.20 ± 11.12% to 33.60 ± 12.68% (mean change 9.40 percentage points; 39% increase; 95% CI: 6.49 to 12.31; *p* < 0.001), Group B from 20.80 ± 7.41% to 51.40 ± 9.56% (mean change 30.60 percentage points; 147% increase; 95% CI: 27.69 to 33.51; *p* < 0.001), and Group C from 21.20 ± 8.22% to 78.60 ± 7.71% (mean change 57.40 percentage points; 271% increase; 95% CI: 54.49 to 60.31; *p* < 0.001) (Table 12).

Between-group comparisons. No significant baseline difference in SFDI was detected among the four groups (*p* = 0.474). At six weeks, SFDI was significantly lower in the control group (25.00 ± 8.89%) than in Groups A (33.60 ± 12.68%; *p* = 0.044), B (51.40 ± 9.56%; *p* < 0.001), and C (78.60 ± 7.71%; *p* < 0.001). Group A had significantly lower scores than Groups B and C, and Group B had significantly lower scores than Group C (all *p* < 0.001) (Table 12).

## 4. Discussion

Interpretation of the primary ITT findings also requires consideration of the post-imputation regression diagnostics. Residual non-normality was frequently flagged for CMAP, latency, and PFDI, while heteroscedasticity was frequently flagged for PFDI and SFDI. The direction and statistical significance of the reported pooled treatment comparisons have not been altered by these diagnostic checks; however, the checks do not establish that conventional regression-based uncertainty estimates are robust to these departures. Accordingly, the estimated between-protocol differences should be interpreted alongside their confidence intervals and the sensitivity of the conclusions to modeling assumptions.

In the primary ITT analysis of all 104 randomized participants, the four treatment groups differed significantly in six-week distal motor latency, CMAP amplitude, PFDI, and SFDI after baseline adjustment and multiple imputation. The Group C complete protocol yielded more favorable outcomes than Control, A, and B for all four outcomes (Bonferroni-adjusted *p* < 0.001 for each Group C comparison). Specifically, compared with Group B, Group C had 0.213 ms lower latency (B − C adjusted MD 0.213 ms; 95% family-wise CI 0.130 to 0.296), 0.553 mV higher CMAP amplitude (B − C MD −0.553 mV; CI −0.648 to −0.457), 24.69 points higher PFDI (B − C MD −24.69; CI −34.29 to −15.08), and 26.66 points higher SFDI (B − C MD −26.66; CI −32.38 to −20.94). Study Groups A and B did not differ significantly for latency or CMAP amplitude after adjustment. The supporting complete-case analyses were broadly concordant in direction, but the primary inference rests on the multiply imputed ITT estimates.

ENoGs are among the most important and reliable methods for assessing the electrophysiological response in acute Bell’s palsy patients. Latency reflects the velocity and responsiveness of nerve conduction, making it a key determinant in the diagnosis of demyelinating lesions [38]. The main variables of ENoG are latency and amplitude. Latency is not as important as amplitude with respect to prognosis in the acute phases of Bell’s palsy [39]. The synchronous discharge of nerve fibers is what the CMAP amplitude represents, and Wallerian degeneration of the nerve is associated mainly with a reduction in the CMAP amplitude. To calculate the degree of nerve degeneration, the amplitude on the affected side is compared with the amplitude on the healthy side [39]. In addition to the occurrence of synkinesis in the late stages of Bell’s palsy, this can be prevented or at least predicted through an important diagnostic tool such as the ENoG amplitude [40].

The improvement observed in the control group in terms of ENoG latency and amplitude may be related to several possible explanations. Most notably, previous studies assumed that within three weeks of the onset of the injury, spontaneous improvement and recovery occurred in 66% to 85% of Bell’s palsy patients, with this rate increasing for up to eight weeks [41,42]. In another study, it was hypothesized that this improvement may be related to the potential impact of facial exercises on facial functions in all stages of Bell’s palsy, whether acute or chronic [9,43]. We cannot overlook the possible contribution of corticosteroids, which are a cornerstone and the first line of defense in acute Bell’s palsy patients. Consequently, the overall improvement in the control group may have been partly related to the timely administration of these medications [3]. This improvement aligns with a previous study that discussed the positive role of adding electrical stimulation to the standard protocol in acute cases of Bell’s palsy and its potential contribution to recovery. Similarly, another study, which is consistent with our findings, demonstrated that patients receiving placebo laser treatment showed significant improvement in the House–Brackmann scale score, thus supporting the potential contribution of the standard protocol to meaningful clinical improvement in acute cases. Furthermore, our results largely agree with another study that compared three groups, including a sham control group, and showed a marked statistical improvement in nerve conduction studies of the facial nerve and the Sunnybrook grading scale (SBGS) among all three groups [44]. This improvement is consistent with the recognized potential for spontaneous recovery in acute Bell’s palsy patients, including those receiving standard treatment at that stage [45].

Since we mentioned the role of standard treatment in the acute phase, one study reported a significant improvement in facial nerve conduction in children with Bell’s palsy when they received standard treatment consisting of steroids and facial exercises [46]. Moreover, one previous study suggested that even an improvement in latency time is a strong indicator that standard conservative treatment is largely sufficient to avoid subsequent surgical interventions such as facial nerve decompression [47].

The greater short-term electrophysiological improvement observed in Group C should be interpreted as an effect of the complete 1064 nm HILT protocol as implemented in this trial rather than as evidence that the 1064 nm wavelength itself is independently superior. The active protocols differed in multiple parameters, including power, delivered energy, treatment duration, application sites, number of treatment points, device characteristics, and mode of delivery. Proposed mechanisms related to deeper tissue penetration and photothermal or photobiomodulatory effects may be biologically relevant [48,49,50,51,52], but these mechanisms and the independent contribution of wavelength were not isolated experimentally in the present study. Previous studies have also reported favorable outcomes with HILT in Bell’s palsy [33,48]; however, comparisons with those studies should likewise be interpreted in the context of differences in complete treatment protocols and outcome measures.

In previous studies, the potential biological effects of high-intensity laser exposure have been discussed in relation to light delivery into deeper tissues, modulation of local inflammatory responses, and processes potentially relevant to peripheral nerve repair [48,49,50,51,52]. Such processes could include changes in cellular metabolism and the tissue environment supporting axonal recovery or remyelination. Nevertheless, neither tissue-level optical dose nor axonal regeneration, myelin repair, or mitochondrial activity was measured in the present trial. These possibilities should therefore be viewed as hypotheses for future mechanistic investigation, not as demonstrated explanations for the superior six-week outcomes of Group C.

An improvement was observed in Group A in ENoG latency and amplitude. Previous experimental work has proposed photobiomodulatory mechanisms involving cytochrome-c oxidase and mitochondrial ATP production, with downstream effects on cellular repair processes [53]. Other preclinical studies have also reported changes in inflammatory signaling and neural repair following 808 nm light exposure [54,55,56,57,58,59,60]. These mechanistic observations provide biological context only; the present trial did not isolate wavelength-specific mechanisms, and the findings for Group A should therefore be interpreted as effects of the complete 808 nm LLLT protocol as implemented.

More specifically, experimental investigations of low-level light exposure have described changes in mitochondrial energy-related pathways and regenerative markers, including growth-associated protein 43 (GAP-43) immunoreactivity in peripheral nerve models [53,54]. Other preclinical work has reported dose-dependent alterations in microglial polarization and neurite growth following 808 nm irradiation [55], alongside favorable observations in experimental nerve-injury studies [56,57,58]. Clinical reports and reviews have also described facial functional responses following near-infrared LLLT, although their designs and dosimetry differ from the current Group A protocol [22,44,59]. The comparatively limited response of Group A on some outcomes might reflect differences in treatment coverage and delivered dose relative to the other active protocols; this remains speculative because individual laser parameters were not independently randomized [60].

Study Group B also showed significant improvement in ENoG outcomes. Previous experimental literature has described biological responses to 980 nm irradiation, including effects on cellular signaling and neural repair [61,62]. However, evidence specifically addressing this protocol in Bell’s palsy remains limited, and the cited mechanistic and preclinical findings should not be interpreted as establishing an independent effect of the 980 nm wavelength. The present findings relate to the complete 980 nm LLLT protocol as implemented in this trial.

The earlier rationale for the 980 nm intervention emphasized wavelength-dependent absorption and the possibility of effects on cell signaling and tissue responses [17,61]. Experimental peripheral nerve work has suggested that 980 nm irradiation may influence repair-related outcomes, but evidence from animal models cannot establish the mechanism or efficacy of the exact protocol used here in human facial nerve palsy [62]. Differences between Groups A and B may additionally reflect treatment-point distribution, total energy delivery, and device settings rather than wavelength alone; the present design cannot disentangle these factors.

We cannot overlook the possible contribution of the facial exercises received by all groups as part of the standard treatment. Several studies have suggested the importance of these exercises at this stage for patients with Bell’s palsy, suggesting that they may help maintain muscle tone in the facial muscles, improve the neurological response of the facial nerve, and protect against muscle wasting or subsequent synkinesis [63]. Therefore, it must be acknowledged that a significant portion of these results may also be related to standard treatment and facial exercises. However, there is still considerable debate regarding the benefits of these exercises in the acute phase of Bell’s palsy [64].

The differing significance decisions for Group A versus sham on PFDI should not be attributed solely to imputation: the ITT and complete-case analyses differ both in included participants and in statistical specification. The ITT comparison is the prespecified primary inferential reference in this report; the complete-case result provides a complementary assessment of consistency rather than an alternative basis for selecting a favorable conclusion.

In the primary ITT analysis, Group C had more favorable PFDI and SFDI outcomes than the other three complete protocols after Bonferroni adjustment. Compared with Group A, Group C was associated with 45.40 points higher PFDI (A − C adjusted MD −45.40; 95% family-wise CI −54.30 to −36.51) and 47.43 points higher SFDI (A − C MD −47.43; CI −52.96 to −41.91). Compared with the sham-control group, the corresponding adjusted differences were 55.75 and 56.06 points in favor of Group C. These results represent protocol-level comparisons under a missing-at-random assumption; they do not isolate wavelength-specific mechanisms or establish long-term benefit.

An important distinction between the primary ITT and supportive complete-case findings concerns the physical Facial Disability Index. In the baseline-adjusted ITT analysis of all 104 randomized participants, Group A had a higher six-week PFDI score than the sham-control group (adjusted mean difference for Control − A, −10.34 points; Bonferroni-adjusted *p* = 0.020; 95% family-wise CI, −19.58 to −1.11). By contrast, the corresponding between-group PFDI comparison was not statistically significant in the 80-participant complete-case analysis (*p* = 0.558). This difference in statistical significance should be interpreted in light of the different analysis populations and model specifications, rather than as evidence of contradictory treatment effects. The revised manuscript prioritizes the ITT estimates, while acknowledging that this particular comparison is less robust across analytical approaches and depends on the missing-at-random assumption.

The sham-control group showed improvements in electrophysiological measures, whereas the supportive complete-case within-group analyses did not detect statistically significant changes in either PFDI (*p* = 0.649) or SFDI (*p* = 0.586). These within-group findings should not be generalized to the primary ITT analysis, which evaluated baseline-adjusted between-group differences rather than within-group change. The apparent divergence between electrophysiological and patient-reported outcomes may reflect differences in the constructs measured and their responsiveness during early recovery. Previous research has also questioned the correspondence between the FDI and clinical measures of facial recovery in acute Bell’s palsy [65]. Nevertheless, the absence of a statistically significant within-group FDI change in the complete-case sham arm does not establish an absence of functional recovery or a lack of benefit from standard care.

The distinction between electrophysiological change and patient-reported facial disability also merits consideration alongside earlier comparative rehabilitation research. A randomized clinical trial comparing low-level laser therapy with electrical stimulation in acute Bell’s palsy examined facial recovery outcomes and provides relevant clinical context for interpreting differences between active laser and non-laser treatment approaches [66]. However, such external comparisons cannot establish that the lack of statistically significant within-group FDI change in our complete-case sham arm was caused by the absence of active laser exposure; spontaneous recovery, concomitant standard care, measurement responsiveness, and the limited sample may all contribute.

Group C, which received HILT, showed the greatest improvement in FDI scores. These results are consistent with several studies, including one that reported tangible changes in facial function with this type of laser compared with standard treatment alone [48]. The study conducted by Al-Alayat et al. corroborates our findings, reporting greater improvement with HILT than with LLLT, massage, and exercise in both subscales of the FDI, where the quantitative data revealed that the HILT intervention had the greatest effect (PFDI from 20.06–39.74). (SFDI from 21.91 to 38.47) after 6 weeks of treatment [33]. A meta-analysis [13] provided strong evidence that laser treatment was associated with improved FDI scores (MD = 17.91, 95% CI; 5.60–30.22) compared with other standard treatments for Bell’s palsy patients.

The larger CMAP amplitude observed at six weeks in Group C may reflect greater short-term electrophysiological recovery under the complete treatment protocol. However, the original percentage improvement and standardized effect sizes were derived from the 80-completer analysis and should not be interpreted as the primary ITT effect estimates. Long-term neural recovery was not assessed.

The primary ITT analysis indicated differences among the four randomized treatment groups for PFDI and SFDI at six weeks, with Group C demonstrating the most favorable outcomes. The supportive complete-case analysis showed a broadly similar ordering. Clinical relevance should be interpreted cautiously because minimal clinically important differences for these particular outcomes and context were not established in this trial.

The findings provide preliminary support for evaluating the complete 1064 nm HILT treatment protocol as an adjunct to standard care in future trials, rather than establishing a preferred wavelength or routine clinical recommendation. The primary ITT comparisons concerned six-week, baseline-adjusted outcomes, with complete-case analyses providing supportive evidence. Treatment protocols varied in wavelength, power, dose, application site, and duration; consequently, the relative contribution of any individual laser parameter cannot be identified. No adverse events were reported during the observed study period, but the sample size and follow-up duration do not establish long-term safety or sustained clinical benefit.

Additional statistical limitations arise from the post-imputation diagnostics: departures from residual normality and evidence of heteroscedasticity, particularly for the FDI outcomes, may affect the calibration of model-based standard errors and confidence intervals. Cook’s distance screening also identified potentially influential observations, although these were retained in the analyses. The fraction of missing information for treatment coefficients (0.179–0.307) indicates non-negligible uncertainty associated with missing follow-up data. Bonferroni correction controlled the six pairwise comparisons within each outcome, but it did not provide study-wide family-wise error control across all four outcomes; CMAP therefore remains the primary outcome, with latency, PFDI, and SFDI interpreted as supportive.

Taken together, these findings warrant further evaluation of the complete Group C protocol as an adjunct to standard care, rather than a recommendation for routine use of a particular laser wavelength. The comparative effects were assessed only at six weeks, and the contribution of individual laser parameters cannot be determined from this design. Electrophysiological outcomes may complement patient-reported measures when monitoring short-term recovery, but longer-term clinical and safety outcomes require further investigation.

Several limitations should be considered. First, the six-week follow-up does not establish the durability of recovery or effects on synkinesis, recurrence, or long-term quality of life. Second, post-treatment House–Brackmann grades were not collected, limiting corroboration of patient-reported and electrophysiological outcomes. Third, this single-center trial may have limited generalizability. Fourth, six-week outcomes were missing for 24 of 104 randomized participants (23.1%). Although 50 multiply imputed datasets allowed their inclusion in the primary ITT analysis, the missing-at-random assumption is unverifiable, and no formal missing-not-at-random sensitivity analysis was reported. Residual non-normality and heteroscedasticity in some imputed-data models may also affect the precision of conventional inference; these results should therefore be interpreted cautiously.

Fifth, although sham procedures and masking measures were implemented, blinding success was not formally assessed; differences in treatment sensation may have introduced expectancy or performance bias. Sixth, Group A was adapted from a case report, and the active protocols differed in wavelength, power, energy delivery, duration, treatment sites, and device characteristics. The trial therefore compares complete intervention protocols and cannot isolate wavelength-specific effects. Seventh, the trial was retrospectively registered (first submitted on 1 April 2026; first publicly posted on 9 April 2026), after enrollment had begun, because of an unintentional administrative oversight and an initial misunderstanding of the requirement for registration before first enrollment. Despite prior institutional ethical approvals, retrospective registration reduces transparency regarding prospectively specified methods and outcomes and is a reporting limitation. Finally, the Facial Disability Index may have limited sensitivity in the acute stage of Bell’s palsy.

## 5. Conclusions

In the primary intention-to-treat analysis of all 104 randomized participants, baseline-adjusted six-week outcomes differed significantly among the sham-control and three active laser therapy protocol groups for CMAP amplitude, distal motor latency, PFDI, and SFDI. The complete 1064 nm HILT protocol showed the most favorable short-term outcomes, and the supportive 80-participant complete-case analysis showed a broadly concordant pattern. Because protocols differed in multiple parameters beyond wavelength, these findings do not establish an independent effect of wavelength. The six-week follow-up and unverifiable missing-at-random assumption limit the strength and generalizability of the conclusions.

Future multicenter randomized controlled trials with longer follow-up periods are warranted to determine whether the observed improvements are sustained over time and to improve generalizability. Standardized protocols are also needed to clarify the contributions of energy dosage, treatment coverage, delivery mode, and wavelength to outcomes in acute Bell’s palsy.

## Figures and Tables

**Figure 1:**
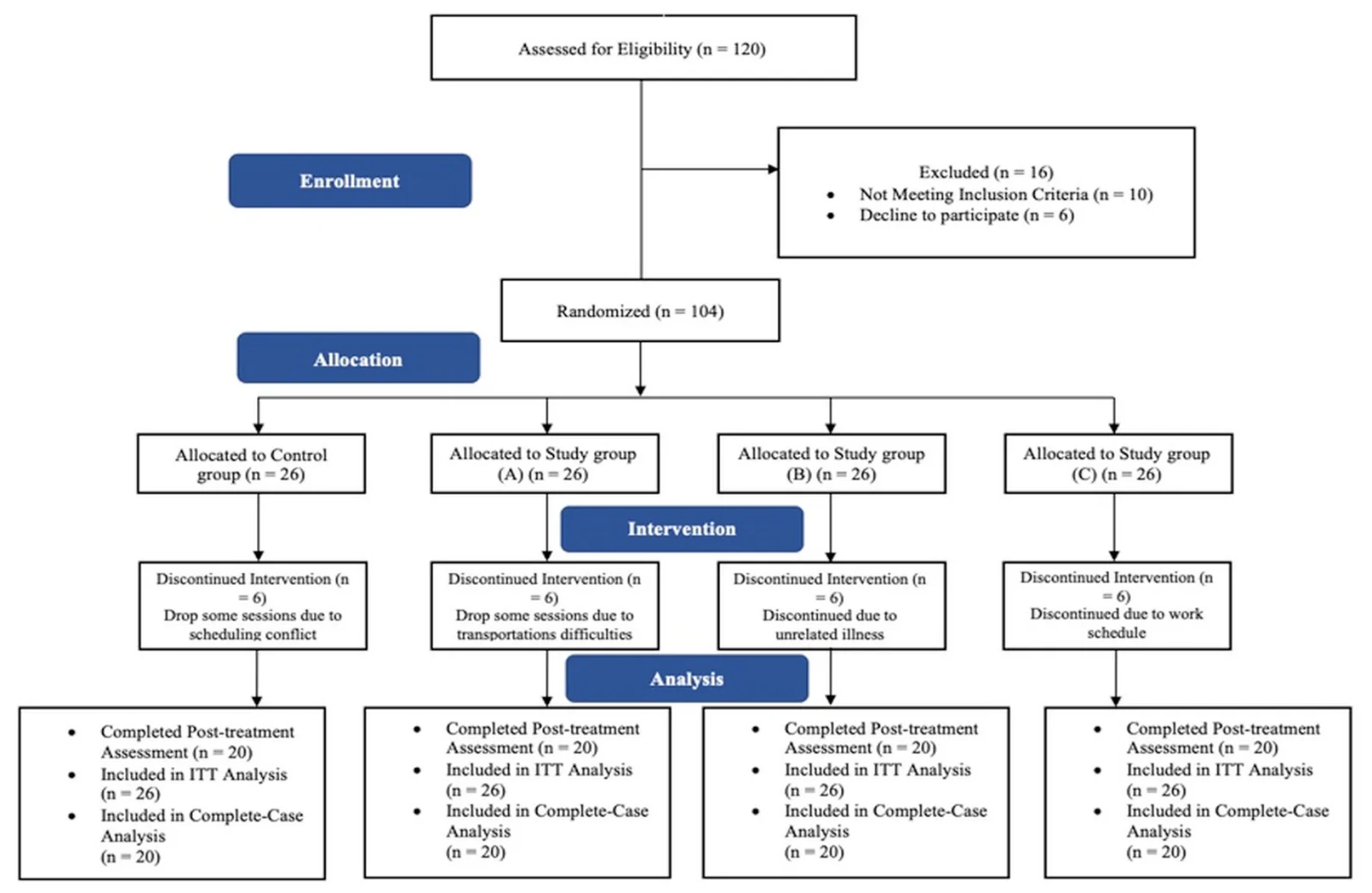
Flow chart for patients in the study.

**Figure 2:**
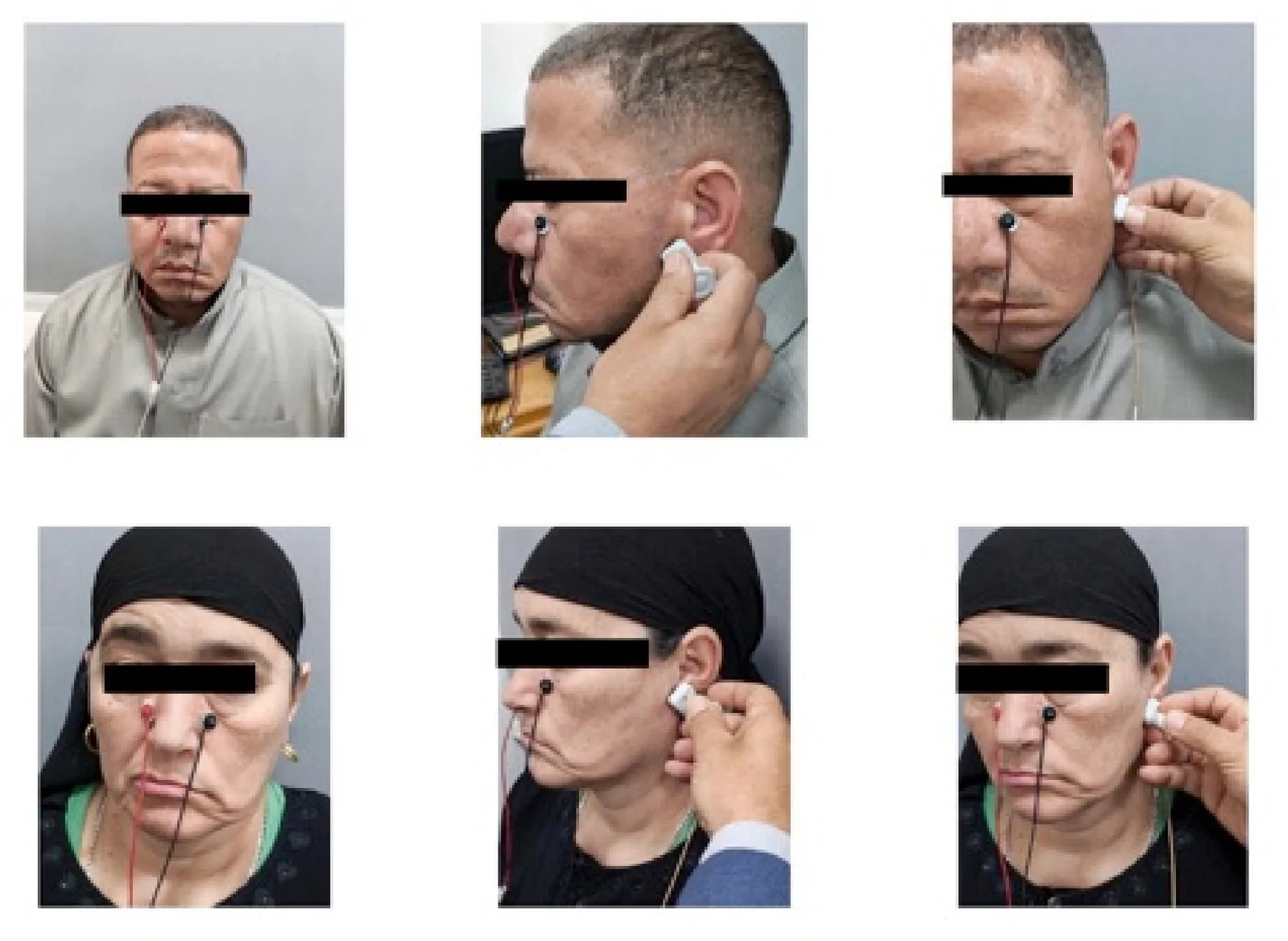
Application of nerve conduction studies in patients with Bell’s palsy.

**Figure 3:**
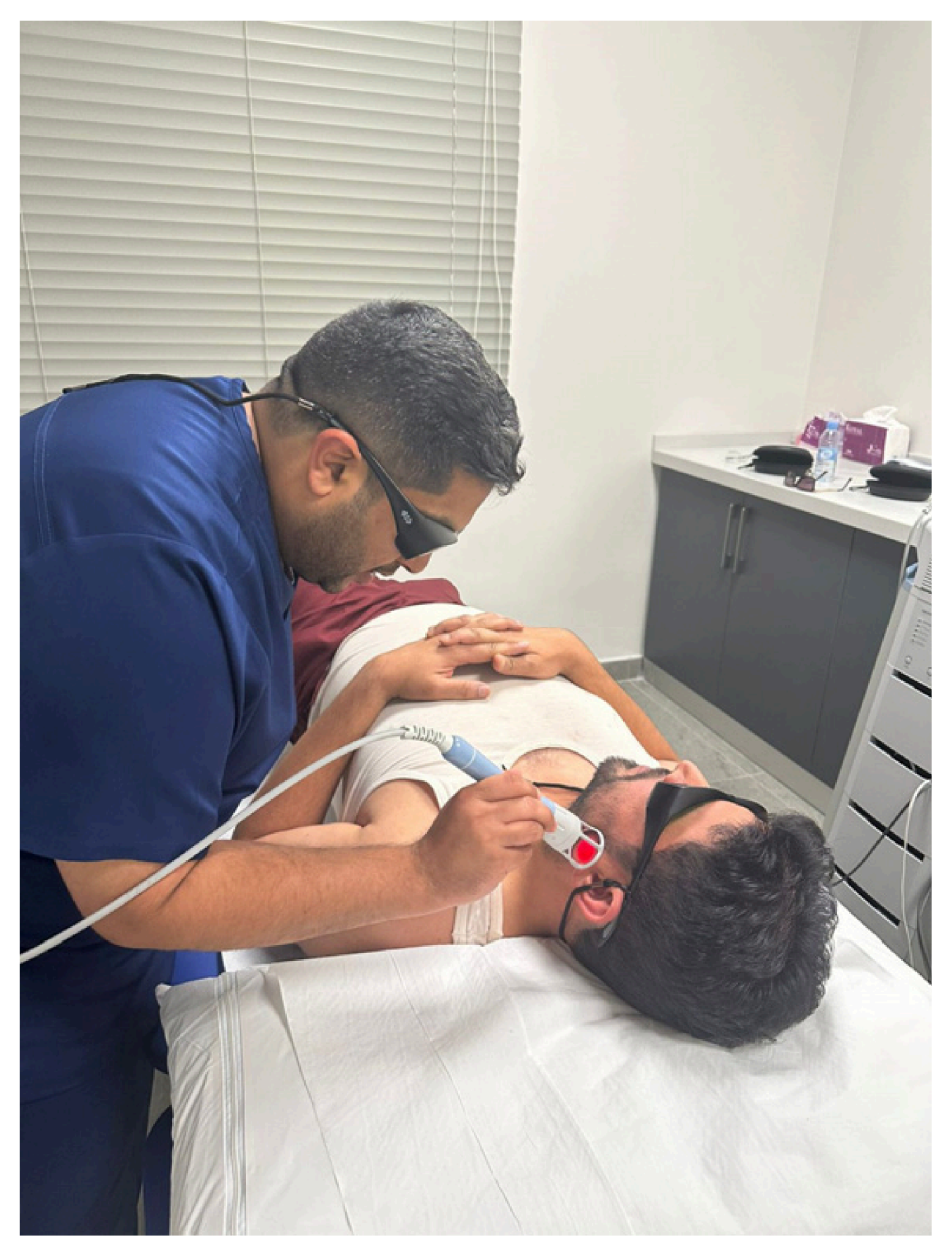
Application of laser therapy on patients with Bell’s palsy.

**Table 1:** Baseline characteristics and exploratory between-group comparisons in all randomized participants (*n* = 104). Continuous variables were compared using one-way ANOVA (F statistics); categorical variables were compared using Pearson χ^2^ tests, with permutation-based *p*-values when expected cell counts were sparse (50,000 permutations).

Variable	Control Group	Study Group A	Study Group B	Study Group C	Statistic	*p*-Value	Significance
Age (years), mean ± SD	39.15 ± 12.77	37.54 ± 11.65	37.81 ± 11.31	38.50 ± 11.52	F(3,100) = 0.098	0.961	Not significant
Onset (hours), mean ± SD	40.91 ± 16.56	40.49 ± 17.45	39.64 ± 17.36	40.03 ± 15.91	F(3,100) = 0.028	0.994	Not significant
Sex (M:F)	12:14	13:13	13:13	13:13	χ^2^(3) = 0.115 (asympt.)	0.990	Not significant
HBGS (III:IV:V:VI)	11:11:3:1	12:10:3:1	12:10:3:1	12:10:3:1	χ^2^(9) = 0.137 (perm.)	1.000	Not significant
Paralysis (Complete:Incomplete)	4:22	4:22	4:22	4:22	χ^2^(3) = 0.000 (perm.)	1.000	Not significant
Corticosteroid use (Yes:No)	26:0	26:0	26:0	26:0	Not estimable	N/A	Not applicable
Antiviral use (Yes:No)	26:0	26:0	26:0	26:0	Not estimable	N/A	Not applicable
Diabetes (Yes:No)	3:23	3:23	3:23	3:23	χ^2^(3) = 0.000 (perm.)	1.000	Not significant
Retroauricular pain (Yes:No)	16:10	15:11	16:10	17:9	χ^2^(3) = 0.325 (asympt.)	0.955	Not significant
Affected side (R:L)	13:13	13:13	13:13	13:13	χ^2^(3) = 0.000 (asympt.)	1.000	Not significant
Not significant							
Baseline CMAP amplitude (mV), mean ± SD	0.64 ± 0.06	0.63 ± 0.07	0.63 ± 0.07	0.61 ± 0.08	F(3,100) = 0.749	0.525	Not significant
Baseline PFDI, mean ± SD	24.62 ± 10.29	22.50 ± 11.77	27.31 ± 9.62	21.73 ± 7.87	F(3,100) = 1.627	0.188	Not significant
Baseline SFDI, mean ± SD	23.23 ± 8.16	23.85 ± 10.58	21.69 ± 7.69	20.62 ± 7.73	F(3,100) = 0.752	0.523	Not significant

**Table 2:** Sex distribution in the four randomized groups (*n* = 104).

Item	Control Group		Study Group A		Study Group B		Study Group C	
	Frequency	Percent	Frequency	Percent	Frequency	Percent	Frequency	Percent
Males	12	46.2%	13	50.0%	13	50.0%	13	50.0%
Females	14	53.8%	13	50.0%	13	50.0%	13	50.0%
Total number of patients	26	100%	26	100%	26	100%	26	100%

**Table 3:** Affected-side distribution in the four randomized groups (*n* = 104).

Item	Control Group		Study Group A		Study Group B		Study Group C	
	Frequency	Percent	Frequency	Percent	Frequency	Percent	Frequency	Percent
Right	13	50.0%	13	50.0%	13	50.0%	13	50.0%
Left	13	50.0%	13	50.0%	13	50.0%	13	50.0%
Total number of patients	26	100%	26	100%	26	100%	26	100%

**Table 4:** Randomized treatment arms and missing six-week assessments (104 randomized; primary ITT population).

Randomized Arm	Randomized (*n*)	Six-Week Assessed (*n*)	Missing at Six Weeks (*n*)	Attrition (%)	Analysis Sets (*n*)
Control	26	20	6	23.1%	ITT 26; complete-case 20
Study Group A (808 nm LLLT)	26	20	6	23.1%	ITT 26; complete-case 20
Study Group B (980 nm)	26	20	6	23.1%	ITT 26; complete-case 20
Study Group C (1064 nm HILT)	26	20	6	23.1%	ITT 26; complete-case 20
Total	104	80	24	23.1%	ITT 104; complete-case 80

**Table 5:** Primary ITT pooled baseline-adjusted pairwise comparisons (50 imputations; Bonferroni-adjusted).

Outcome	Contrast (First − Second)	MD	95% Family-Wise CI	*p*	MI df
Latency (ms)	Control − A	0.295	[0.213, 0.377]	<0.001	68.4
Latency (ms)	Control − B	0.269	[0.187, 0.350]	<0.001	68.1
Latency (ms)	Control − C	0.482	[0.404, 0.559]	<0.001	78.4
Latency (ms)	A − B	−0.027	[−0.109, 0.056]	1.000	67.2
Latency (ms)	A − C	0.187	[0.107, 0.267]	<0.001	72.8
Latency (ms)	B − C	0.213	[0.130, 0.296]	<0.001	65.3
CMAP amplitude (mV)	Control − A	−0.918	[−1.006, −0.830]	<0.001	71.4
CMAP amplitude (mV)	Control − B	−0.882	[−0.971, −0.793]	<0.001	69.8
CMAP amplitude (mV)	Control − C	−1.435	[−1.529, −1.341]	<0.001	62.2
CMAP amplitude (mV)	A − B	0.036	[−0.053, 0.125]	1.000	70.1
CMAP amplitude (mV)	A − C	−0.517	[−0.608, −0.426]	<0.001	66.1
CMAP amplitude (mV)	B − C	−0.553	[−0.648, −0.457]	<0.001	58.2
PFDI	Control − A	−10.344	[−19.580, −1.107]	0.020	64.9
PFDI	Control − B	−31.061	[−40.322, −21.800]	<0.001	64.8
PFDI	Control − C	−55.747	[−64.567, −46.927]	<0.001	73.8
PFDI	A − B	−20.718	[−29.877, −11.558]	<0.001	68.5
PFDI	A − C	−45.404	[−54.300, −36.507]	<0.001	71.2
PFDI	B − C	−24.686	[−34.293, −15.079]	<0.001	61.3
SFDI	Control − A	−8.626	[−14.211, −3.042]	<0.001	73.1
SFDI	Control − B	−29.401	[−35.227, −23.575]	<0.001	65.7
SFDI	Control − C	−56.061	[−61.804, −50.319]	<0.001	69.0
SFDI	A − B	−20.775	[−26.782, −14.767]	<0.001	61.0
SFDI	A − C	−47.435	[−52.955, −41.914]	<0.001	77.1
SFDI	B − C	−26.660	[−32.378, −20.943]	<0.001	68.8

**Table 6:** Primary ITT pooled omnibus treatment-group effects (50 imputations).

Outcome	ITT Pooled Wald χ^2^	df	Asymptotic *p*	Analysis Population	Role
Latency	284.90	3	<0.001	104 randomized	Primary ITT
CMAP amplitude	1844.49	3	<0.001	104 randomized	Primary ITT
PFDI	359.44	3	<0.001	104 randomized	Primary ITT
SFDI	842.37	3	<0.001	104 randomized	Primary ITT

**Table 7:** Sensitivity Analysis.

Outcome	Contrast	ITT Adjusted MD	CC Adjusted MD	CC − ITT	CC 95% FW CI	CC Adj. *p*
CMAP	Control − A	−0.918	−0.920	−0.002	[−1.006, −0.834]	<0.001
CMAP	Control − B	−0.882	−0.885	−0.003	[−0.971, −0.799]	<0.001
CMAP	Control − C	−1.435	−1.436	−0.001	[−1.523, −1.349]	<0.001
CMAP	A − B	0.036	0.035	−0.001	[−0.051, 0.121]	1.000
CMAP	A − C	−0.517	−0.516	+0.001	[−0.603, −0.429]	<0.001
CMAP	B − C	−0.553	−0.551	+0.002	[−0.637, −0.464]	<0.001
Latency	Control − A	0.295	0.290	−0.005	[0.211, 0.369]	<0.001
Latency	Control − B	0.269	0.265	−0.004	[0.186, 0.344]	<0.001
Latency	Control − C	0.482	0.479	−0.003	[0.400, 0.557]	<0.001
Latency	A − B	−0.027	−0.025	+0.002	[−0.104, 0.054]	1.000
Latency	A − C	0.187	0.189	+0.002	[0.110, 0.267]	<0.001
Latency	B − C	0.213	0.214	+0.001	[0.135, 0.292]	<0.001
PFDI	Control − A	−10.344	−10.191	+0.153	[−18.648, −1.734]	0.010
PFDI	Control − B	−31.061	−30.827	+0.234	[−39.304, −22.350]	<0.001
PFDI	Control − C	−55.747	−55.905	−0.158	[−64.408, −47.403]	<0.001
PFDI	A − B	−20.718	−20.636	+0.082	[−29.218, −12.054]	<0.001
PFDI	A − C	−45.404	−45.715	−0.311	[−54.152, −37.277]	<0.001
PFDI	B − C	−24.686	−25.079	−0.393	[−33.750, −16.407]	<0.001
SFDI	Control − A	−8.626	−8.600	+0.026	[−14.092, −3.108]	<0.001
SFDI	Control − B	−29.401	−29.245	+0.156	[−34.788, −23.702]	<0.001
SFDI	Control − C	−56.061	−56.110	−0.049	[−61.642, −50.578]	<0.001
SFDI	A − B	−20.775	−20.645	+0.130	[−26.188, −15.102]	<0.001
SFDI	A − C	−47.435	−47.510	−0.075	[−53.042, −41.978]	<0.001
SFDI	B − C	−26.660	−26.865	−0.205	[−32.358, −21.372]	<0.001

**Table 8:** (**A**). Statistical assumption checks for the four outcomes at baseline and post-treatment. (**B**). Results of the mixed-design multivariate analysis of variance.

(**A**)					
**Outcome/time**	Shapiro–Wilk *p* range *	**Groups with *p* ≥ 0.05**	**Levene F**	**Levene *p***	**Variance homogeneity**
Latency—Pre	0.0056–0.0063	0/4	0.012	0.998	Supported
Latency—Post	<0.001–0.0168	0/4	0.655	0.583	Supported
Amplitude—Pre	<0.001–0.0012	0/4	0.175	0.913	Supported
Amplitude—Post	0.0009–0.0110	0/4	0.321	0.810	Supported
PFDI—Pre	0.0281–0.1963	3/4	1.325	0.272	Supported
PFDI—Post	0.0232–0.3360	2/4	1.956	0.128	Supported
SFDI—Pre	0.1828–0.8189	4/4	1.357	0.262	Supported
SFDI—Post	0.1206–0.8210	4/4	2.214	0.093	Supported
(**B**)					
**Mixed MANOVA**					
**Effect**		**Pillai’s Trace**	**F (df1, df2)**	**Partial η^2^**	***p*** **-value**
Effect of treatment (group effect)		1.336	15.056 (12, 225)	0.445	<0.001
Effect of time		0.995	3676.630 (4, 73)	0.995	<0.001
Interaction effect (treatment × time)		1.735	25.720 (12, 225)	0.578	<0.001

* Shapiro–Wilk *p*-value range across the four randomized groups. A *p*-value < 0.05 indicates evidence of departure from normality. Levene’s test assessed homogeneity of variance. Box’s M was evaluated separately for the four-dimensional outcome vector at baseline and post-treatment.

**Table 9:** Effect of treatment on latency (supportive complete-case analysis; *n* = 80).

**Latency**					
**Mixed ANOVA**					
	**F-value**	**Partial (η^2^)**	***p*** **-value**		
Interaction effect	49.927	0.663	<0.001		
Time effect	4668.573	0.984	<0.001		
Group effect	61.54	0.708	<0.001		
**Within-group Comparison (time effect)**					
**Group**	**Control group (*n* = 20)**	**Study group A (*n* = 20)**	**Study group B (*n* = 20)**	**Study group C (*n* = 20)**	
Pre	4.82 ± 0.08	4.82 ± 0.08	4.82 ± 0.08	4.83 ± 0.07	
Post	4.11 ± 0.08	3.82 ± 0.12	3.85 ± 0.06	3.63 ± 0.09	
MD	−0.71	−1.00	−0.97	−1.20	
% of change	15%	21%	20%	25%	
95% CI	[−0.764, −0.651]	[−1.054, −0.941]	[−1.029, −0.916]	[−1.251, −1.139]	
Cohen’s dz (paired)		5.48	7.08	9.42	9.14
*p*-value	<0.001	<0.001	<0.001	<0.001	
Significance	S	S	S	S	
**Between-group Comparison (group effect)**					
**Time**		**MD**	**95% CI**	***p*** **-value**	**Significance**
Pre	Control vs. A	0	[−0.065, 0.065]	1	NS
	Control vs. B	0	[−0.065, 0.065]	1	NS
	Control vs. C	−0.008	[−0.072, 0.057]	1	NS
	A vs. B	0	[−0.065, 0.065]	1	NS
	A vs. C	−0.008	[−0.072, 0.057]	1	NS
	B vs. C	−0.008	[−0.072, 0.057]	1	NS
Post	Control vs. A	0.29	[0.211, 0.369]	<0.001	S
	Control vs. B	0.27	[0.186, 0.344]	<0.001	S
	Control vs. C	0.48	[0.401, 0.559]	<0.001	S
	A vs. B	−0.025	[−0.104, 0.054]	1	NS
	A vs. C	0.19	[0.111, 0.269]	<0.001	S
	B vs. C	0.215	[0.136, 0.294]	<0.001	S

Values were calculated directly from the paired data (*n* = 20 per group). Pairwise between-group *p*-values and 95% CIs are Bonferroni-adjusted for the six pairwise comparisons within this outcome; the treatment × time interaction remained significant under the four-outcome Bonferroni threshold (α = 0.0125).

**Table 10:** Effect of treatment on amplitude (supportive complete-case analysis; *n* = 80).

**Amplitude**					
**Mixed ANOVA**					
	**F-value**	**Partial (η^2^)**	***p*** **-value**		
Interaction effect	549.731	0.956	<0.001		
Time effect	10,899.685	0.993	<0.001		
Group effect	487.804	0.951	<0.001		
**Within-group Comparison (time effect)**					
**Group**	**Control group (*n* = 20)**	**Study group A (*n* = 20)**	**Study group B (*n* = 20)**	**Study group C (*n* = 20)**	
Pre	0.65 ± 0.05	0.64 ± 0.06	0.64 ± 0.06	0.62 ± 0.07	
Post	1.18 ± 0.11	2.10 ± 0.08	2.06 ± 0.09	2.61 ± 0.11	
MD	0.53	1.46	1.42	1.99	
% of change	82%	228%	222%	321%	
95% CI	[0.478, 0.582]	[1.403, 1.507]	[1.373, 1.477]	[1.943, 2.047]	
**Cohen’s dz (paired)**		4.90	14.57	11.02	16.16
*p*-value	<0.001	<0.001	<0.001	<0.001	
Significance	S	S	S	S	
**Between-group Comparison (group effect)**					
**Time**		**MD**	**95% CI**	***p*** **-value**	**Significance**
Pre	Control vs. A	0.005	[−0.048, 0.058]	1	NS
	Control vs. B	0.01	[−0.043, 0.063]	1	NS
	Control vs. C	0.03	[−0.023, 0.083]	0.767	NS
	A vs. B	0.005	[−0.048, 0.058]	1	NS
	A vs. C	0.025	[−0.028, 0.078]	1	NS
	B vs. C	0.02	[−0.033, 0.073]	1	NS
Post	Control vs. A	−0.92	[−1.005, −0.835]	<0.001	S
	Control vs. B	−0.88	[−0.97, −0.799]	<0.001	S
	Control vs. C	−1.43	[−1.52, −1.349]	<0.001	S
	A vs. B	0.04	[−0.05, 0.12]	1	NS
	A vs. C	−0.51	[−0.6, −0.429]	<0.001	S
	B vs. C	−0.55	[−0.635, −0.465]	<0.001	S

Values were calculated directly from the paired data (*n* = 20 per group). Pairwise between-group *p*-values and 95% CIs are Bonferroni-adjusted for the six pairwise comparisons within this outcome; the treatment × time interaction remained significant under the four-outcome Bonferroni threshold (α = 0.0125).

**Table 11:** Effect of treatment on the PFDI (supportive complete-case analysis; *n* = 80).

**Physical Facial Disability Index (PFDI) (%)**					
**Mixed ANOVA**					
	**F-value**	**Partial (η^2^)**	***p*** **-value**		
Interaction effect	128.063	0.835	<0.001		
Time effect	534.121	0.875	<0.001		
Group effect	20.067	0.442	<0.001		
**Within-group Comparison (time effect)**					
**Group**	**Control group (*n* = 20)**	**Study group A (*n* = 20)**	**Study group B (*n* = 20)**	**Study group C (*n* = 20)**	
Pre	24.75 ± 11.06	22.25 ± 13.13	28 ± 10.05	20.75 ± 8.63	
Post	25.75 ± 10.92	33.5 ± 17.18	59.75 ± 16.58	77.75 ± 11.86	
MD	1.00	11.25	31.75	57.00	
% of change	4%	51%	113%	275%	
95% CI	[−3.352, 5.352]	[6.898, 15.602]	[27.398, 36.102]	[52.648, 61.352]	
**Cohen’s dz (paired)**		0.49	2.10	2.91	3.76
*p*-value	0.649	<0.001	<0.001	<0.001	
Significance	NS	S	S	S	
**Between-group Comparison (group effect)**					
**Time**		**MD**	**95% CI**	***p*** **-value**	**Significance**
Pre	Control vs. A	2.5	[−6.79, 11.79]	1	NS
	Control vs. B	−3.25	[−12.54, 6.04]	1	NS
	Control vs. C	4	[−5.29, 13.29]	1	NS
	A vs. B	−5.75	[−15.04, 3.54]	0.586	NS
	A vs. C	1.5	[−7.79, 10.79]	1	NS
	B vs. C	7.25	[−2.04, 16.54]	0.226	NS
Post	Control vs. A	−7.75	[−20.09, 4.59]	0.558	NS
	Control vs. B	−34	[−46.34, −21.66]	<0.001	S
	Control vs. C	−52	[−64.34, −39.66]	<0.001	S
	A vs. B	−26.25	[−38.59, −13.91]	<0.001	S
	A vs. C	−44.25	[−56.59, −31.91]	<0.001	S
	B vs. C	−18	[−30.34, −5.66]	0.001	S

Values were calculated directly from the paired data (*n* = 20 per group). Pairwise between-group *p*-values and 95% CIs are Bonferroni-adjusted for the six pairwise comparisons within this outcome; the treatment × time interaction remained significant under the four-outcome Bonferroni threshold (α = 0.0125).

**Table 12:** Effect of treatment on the social facial disability index (SFDI) (supportive complete-case analysis; *n* = 80).

**Social Facial Disability Index (SFDI) (%)**					
**Mixed ANOVA**					
	**F-value**	**Partial (η^2^)**	***p*** **-value**		
Interaction effect	298.06	0.922	<0.001		
Time effect	1129.046	0.937	<0.001		
Group effect	31.14	0.551	<0.001		
**Within-group Comparison (time effect)**					
**Group**	**Control group (*n* = 20)**	**Study group A (*n* = 20)**	**Study group B (*n* = 20)**	**Study group C (*n* = 20)**	
Pre	24.2 ± 8.94	24.2 ± 11.12	20.8 ± 7.41	21.2 ± 8.22	
Post	25 ± 8.89	33.6 ± 12.68	51.4 ± 9.56	78.6 ± 7.71	
MD	0.8	9.4	30.6	57.4	
% of change	3%	39%	147%	271%	
95% CI	[−2.11, 3.71]	[6.49, 12.31]	[27.69, 33.51]	[54.49, 60.31]	
**Cohen’s dz (paired)**		0.49	2.16	4.03	6.00
*p*-value	0.586	<0.001	<0.001	<0.001	
Significance	NS	S	S	S	
**Between-group Comparison (group effect)**					
**Time**		**MD**	**95% CI**	***p*** **-value**	**Significance**
Pre	Control vs. A	0	[−7.74, 7.74]	1	NS
	Control vs. B	3.4	[−4.34, 11.14]	1	NS
	Control vs. C	3	[−4.74, 10.74]	1	NS
	A vs. B	3.4	[−4.34, 11.14]	1	NS
	A vs. C	3	[−4.74, 10.74]	1	NS
	B vs. C	−0.4	[−8.14, 7.34]	1	NS
Post	Control vs. A	−8.6	[−17.06, −0.14]	0.044	S
	Control vs. B	−26.4	[−34.86, −17.94]	<0.001	S
	Control vs. C	−53.6	[−62.06, −45.14]	<0.001	S
	A vs. B	−17.8	[−26.26, −9.34]	<0.001	S
	A vs. C	−45	[−53.46, −36.54]	<0.001	S
	B vs. C	−27.2	[−35.66, −18.74]	<0.001	S

Values were calculated directly from the paired data (*n* = 20 per group). Pairwise between-group *p*-values and 95% CIs are Bonferroni-adjusted for the six pairwise comparisons within this outcome; the treatment × time interaction remained significant under the four-outcome Bonferroni threshold (α = 0.0125).

## Data Availability

All the data generated or analyzed during this study are available from the corresponding author upon reasonable request.

## Supplementary Materials

The following supporting information can be downloaded at: https://www.mdpi.com/article/10.3390/jcm15208113/s1, Supplementary Materials: Supplementary Figures S1–S12 present Q–Q plots, residual-versus-fitted plots, and Cook’s distance plots for distal motor latency, CMAP amplitude, PFDI, and SFDI in the primary ITT analysis (representative imputation 1 of 50). Supplementary Figures S13–S20 present descriptive complete-case figures for latency, CMAP amplitude, PFDI, and SFDI. Figure legends: Figure S1. Distal motor latency: Q–Q plot. Figure S2. Distal motor latency: residuals versus fitted values. Figure S3. Distal motor latency: Cook’s distance. Figure S4. CMAP amplitude: Q–Q plot. Figure S5. CMAP amplitude: residuals versus fitted. Figure S6. CMAP amplitude: Cook’s distance. Figure S7. PFDI: Q–Q plot. Figure S8. PFDI: residuals versus fitted values. Figure S9. PFDI: Cook’s distance. Figure S10. SFDI: Q–Q plot. Figure S11. SFDI: residuals versus fitted values. Figure S12. SFDI: Cook’s distance. Figure S13. Mean latency values pre- and posttreatment in all groups. Figure S14. Mean latency values pre- and posttreatment among the groups. Figure S15. CMAP amplitude values pre- and posttreatment in all groups. Figure S16. CMAP amplitude values pre- and posttreatment among the groups. Figure S17. Mean SFDI values pre- and posttreatment in all groups. Figure S18. Mean PFDI values pre- and posttreatment among the groups. Figure S19. Mean SFDI values pre- and posttreatment in all groups. Figure S20. Mean SFDI values pre- and posttreatment among the groups.

## Protocol Accessibility

The publicly accessible trial registration record is available at ClinicalTrials.gov (ID: NCT07519421) and can be obtained upon reasonable request from the corresponding author (pt.ahmad3@gmail.com).

## Abbreviations

LLLT	Low-level laser therapy
HILT	High-intensity laser therapy
Nd:YAG	Neodymium-doped yttrium aluminum garnet
CMAP	Compound muscle action potential
FDI	Facial Disability Index
PFDI	Physical Facial Disability Index
SFDI	Social Facial Disability Index
ATP	Adenosine triphosphate
ROS	Reactive oxygen species
ANOVA	Analysis of variance
MANOVA	Multivariate analysis of variance
