# Supplementary material for: Comparative Effects of Three Active Laser Therapy Protocols and a Sham Protocol on Neurophysiological and Functional Outcomes in Patients with Bell’s Palsy: A Four-Arm Randomized Controlled Trial
Source: J Clin Med. 2026 Oct 19;15(20):8113. doi: 10.3390/jcm15208113 (PMC13706096; doi:10.3390/jcm15208113)
Supplement: Supplementary file 1 [file jcm-15-08113-s001.zip › jcm-4579548-supplementary.pdf]

Based on the retained 50 imputed outcome matrices used to reproduce the pre-existing ITT estimates. Figures show representative imputation 1 of 50; numerical diagnostic counts in the manuscript summarize all 50 imputations. These are diagnostic illustrations, not additional treatment-effect analyses.

Figure S1. Distal motor latency: Q–Q plot

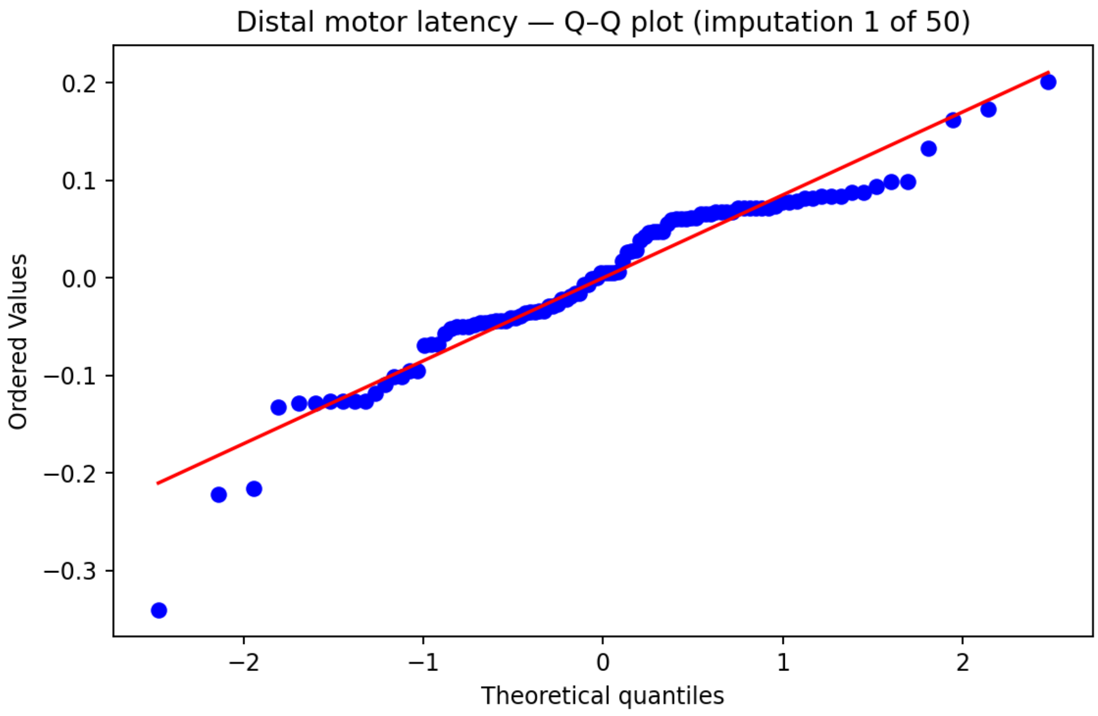

Figure S1 : six-week outcome regressed on randomized group and corresponding baseline outcome; representative completed dataset 1/50.

Figure S2. Distal motor latency: Residuals versus fitted

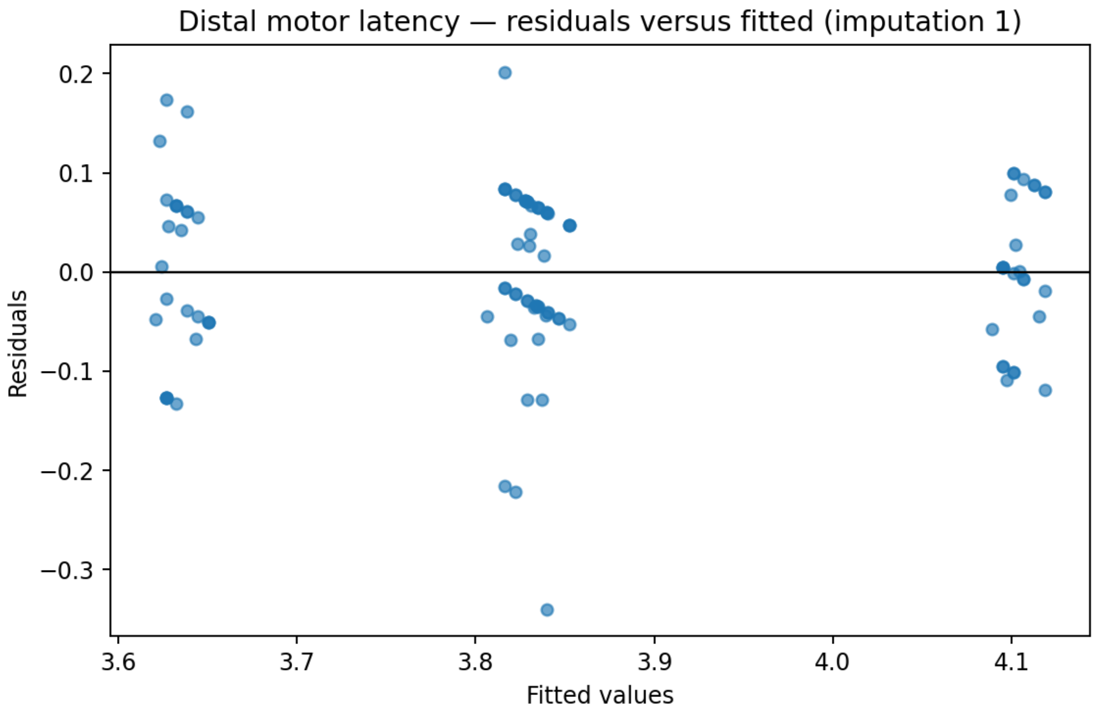

Figure S2 : six-week outcome regressed on randomized group and corresponding baseline outcome; representative completed dataset 1/50.

Figure S3. Distal motor latency: Cook's distance

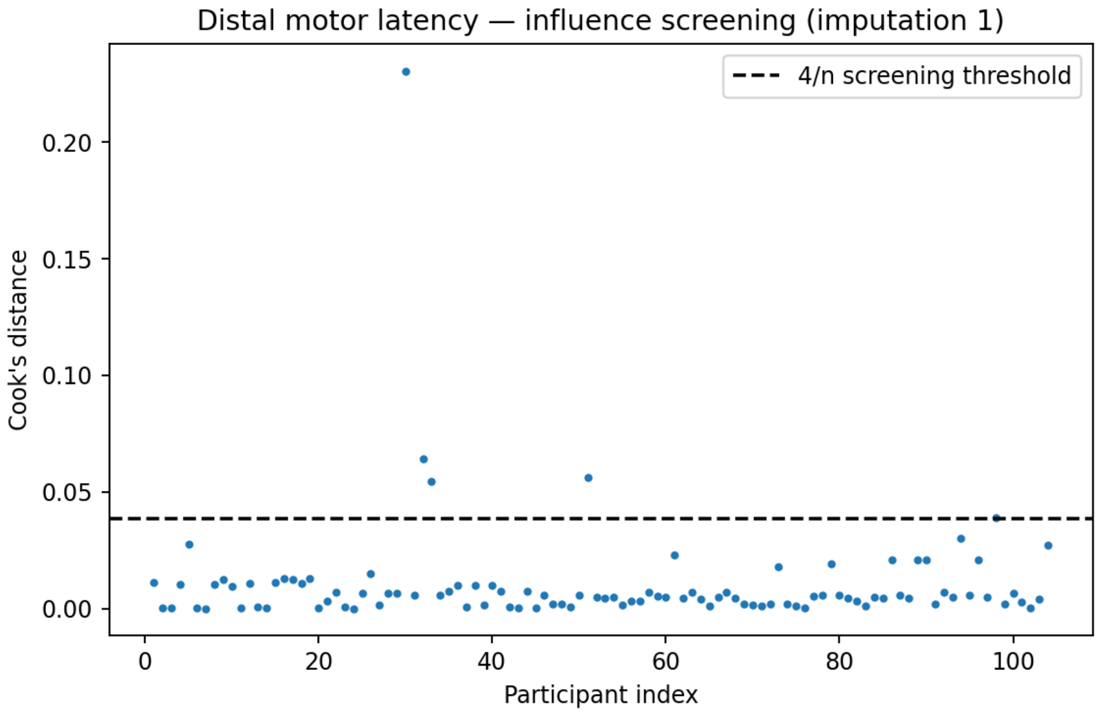

Figure S3 : six-week outcome regressed on randomized group and corresponding baseline outcome; representative completed dataset 1/50.

Figure S4. CMAP amplitude: Q–Q plot

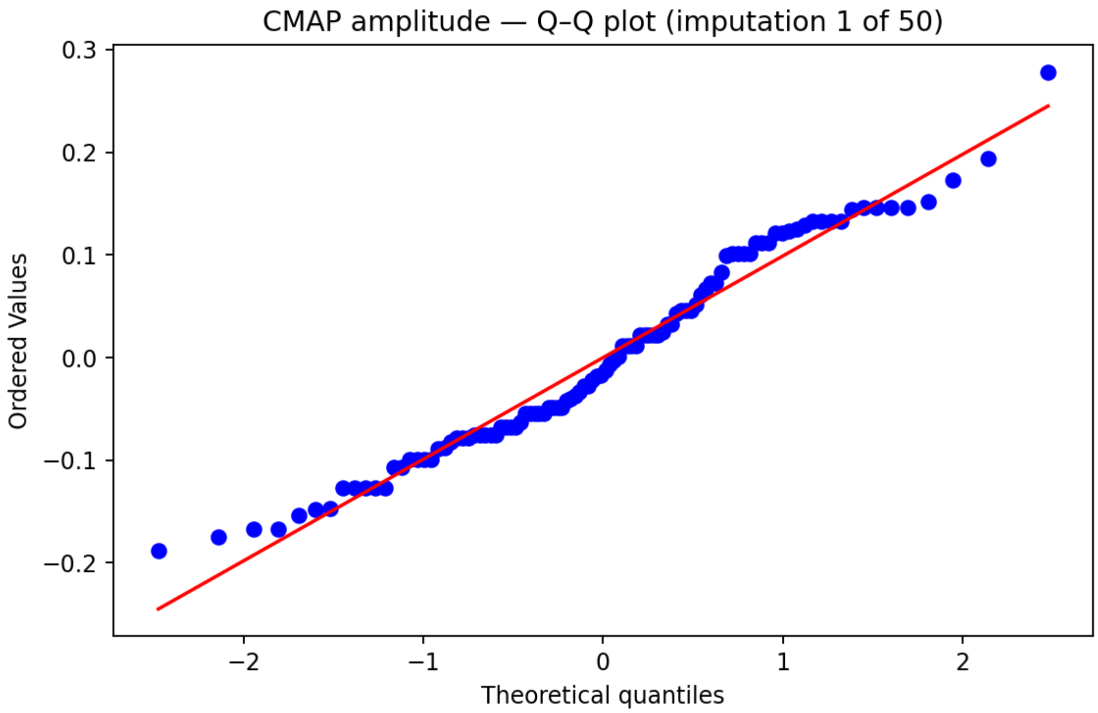

Figure S4 : six-week outcome regressed on randomized group and corresponding baseline outcome; representative completed dataset 1/50.

Figure S5. CMAP amplitude: Residuals versus fitted

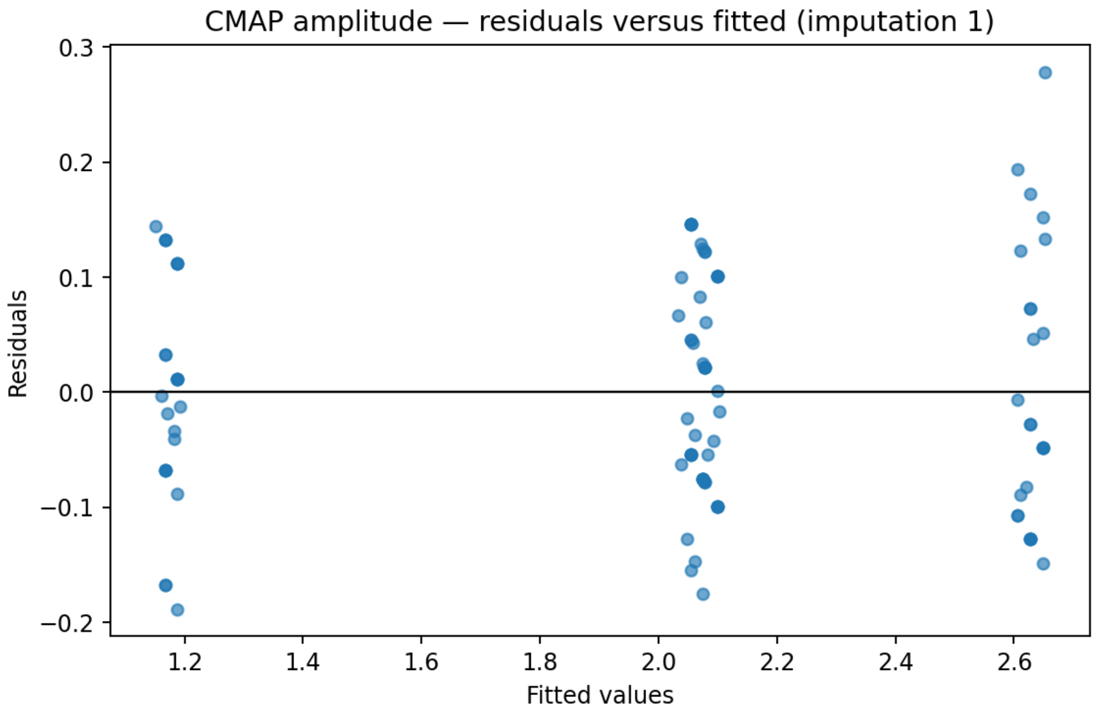

Figure S5 : six-week outcome regressed on randomized group and corresponding baseline outcome; representative completed dataset 1/50.

Figure S6. CMAP amplitude: Cook's distance

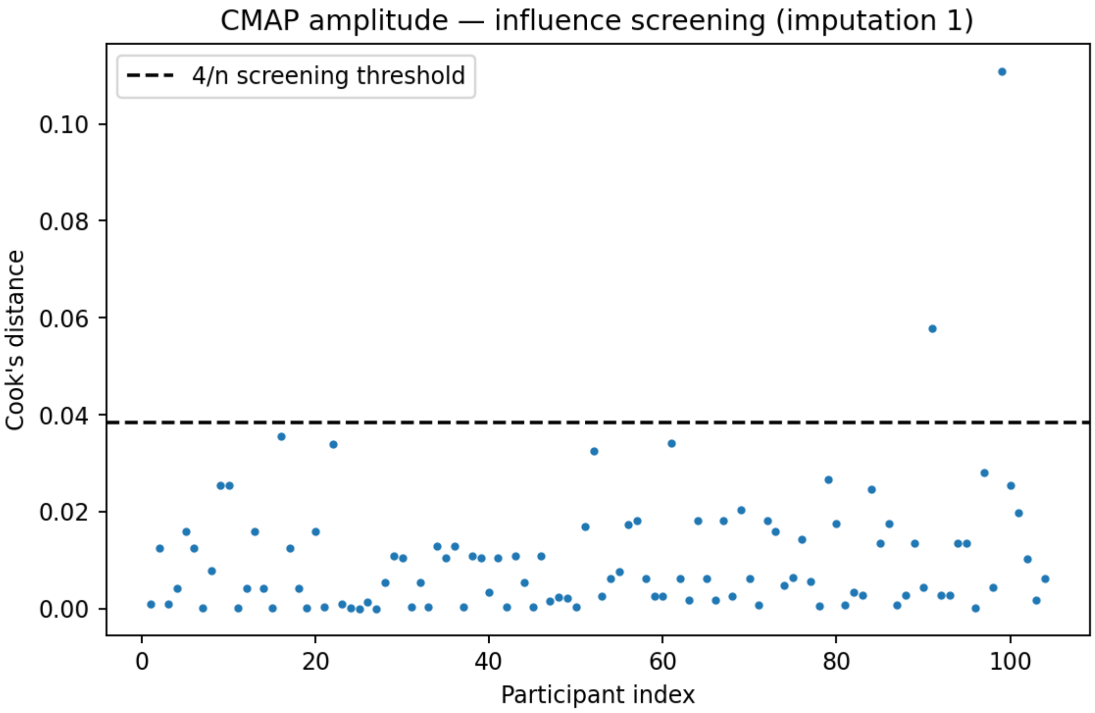

Figure S6 : six-week outcome regressed on randomized group and corresponding baseline outcome; representative completed dataset 1/50.

Figure S7. PFDI: Q–Q plot

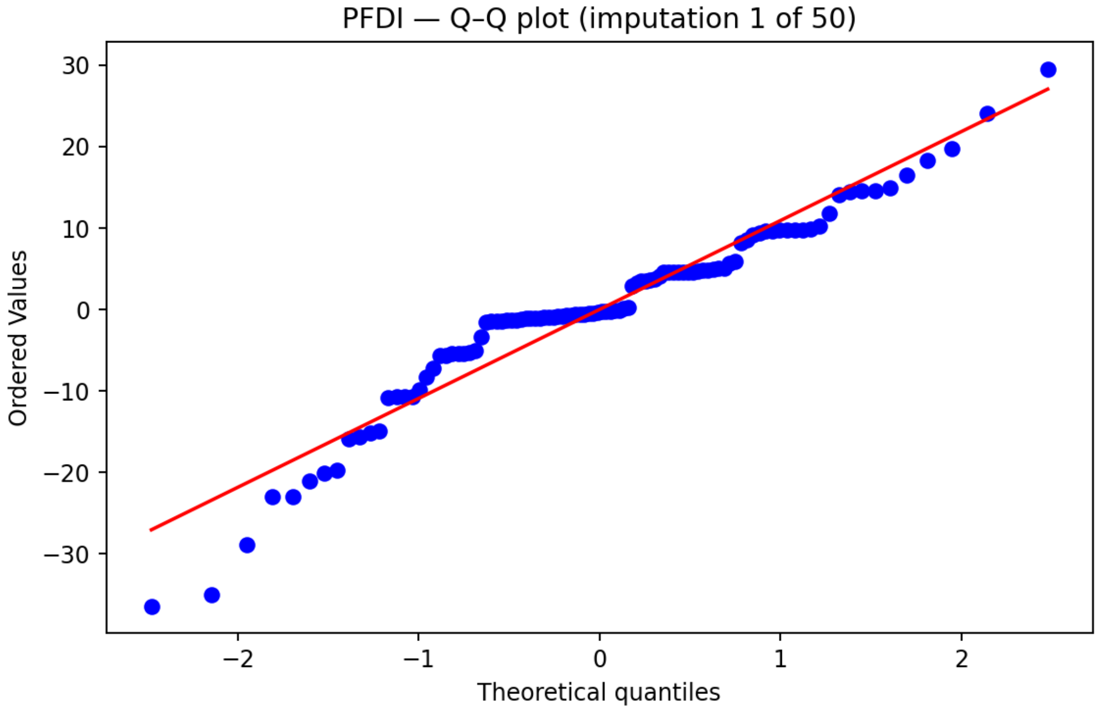

Figure S7 : six-week outcome regressed on randomized group and corresponding baseline outcome; representative completed dataset 1/50.

Figure S8. PFDI: Residuals versus fitted

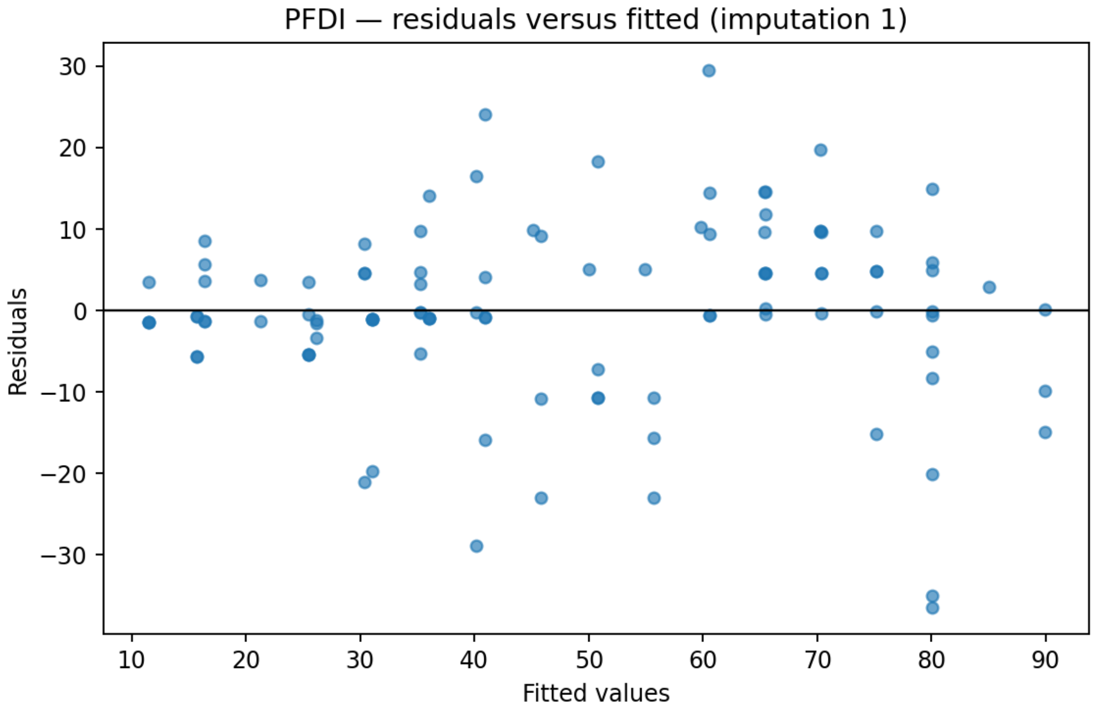

Figure S8 : six-week outcome regressed on randomized group and corresponding baseline outcome; representative completed dataset 1/50.

Figure S9. PFDI: Cook's distance

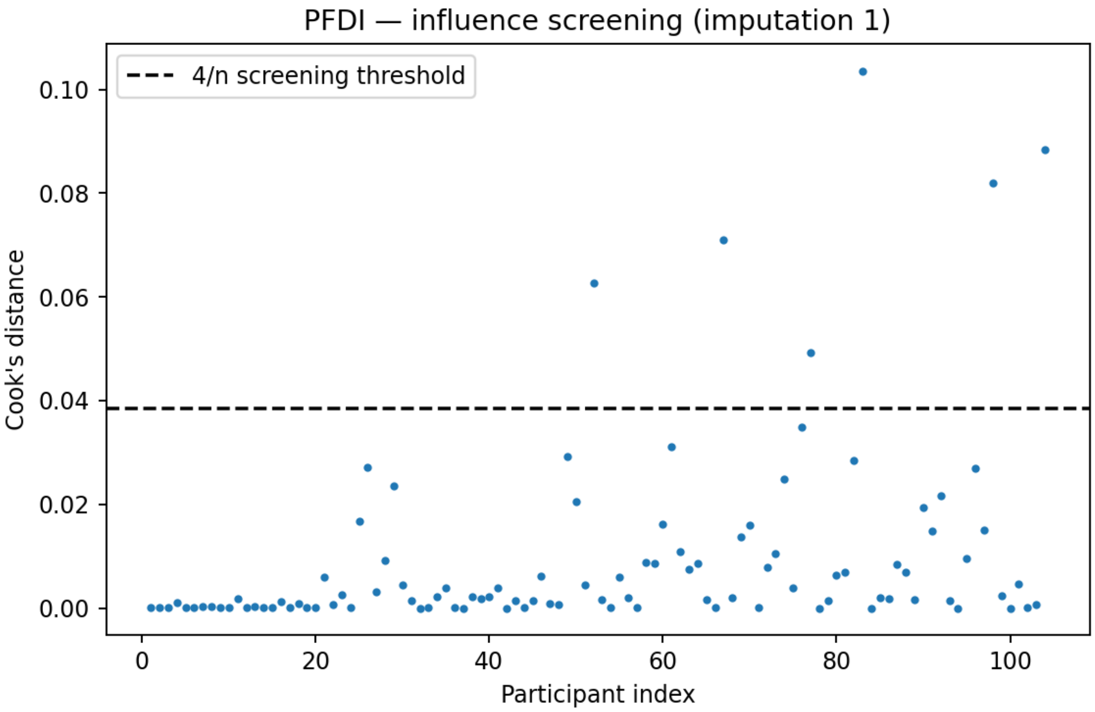

Figure S9 : six-week outcome regressed on randomized group and corresponding baseline outcome; representative completed dataset 1/50.

Figure S10. SFDI: Q–Q plot

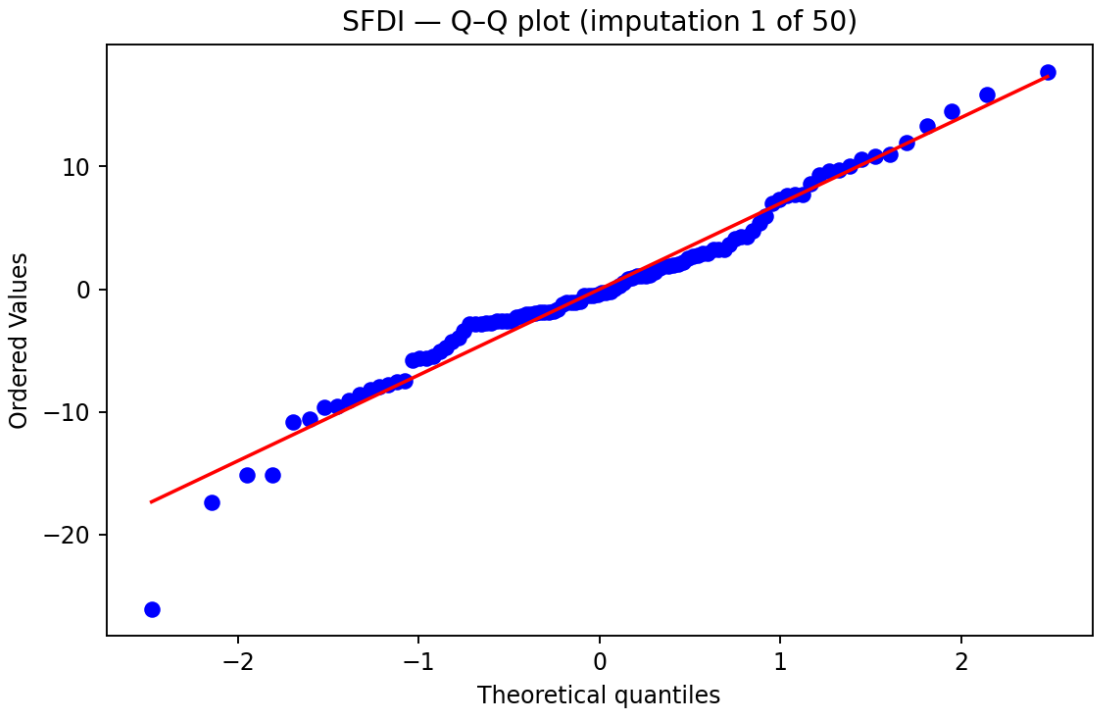

Figure S10 : six-week outcome regressed on randomized group and corresponding baseline outcome; representative completed dataset 1/50.

Figure S11. SFDI: Residuals versus fitted

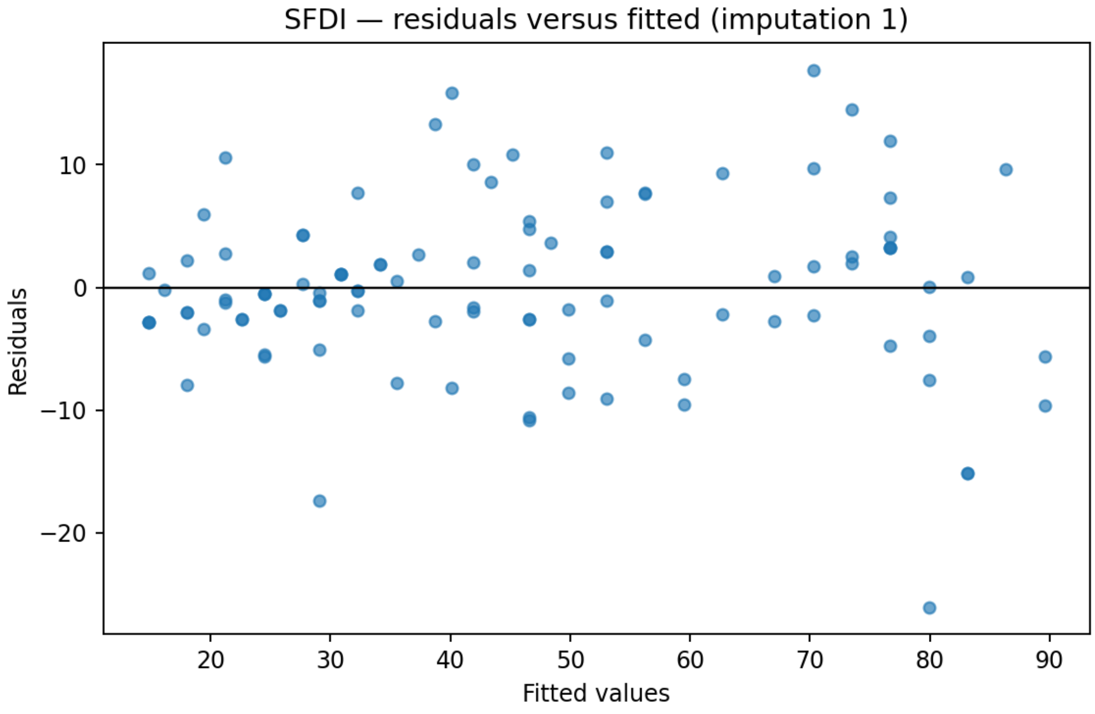

Figure S11 : six-week outcome regressed on randomized group and corresponding baseline outcome; representative completed dataset 1/50.

Figure S12. SFDI: Cook's distance

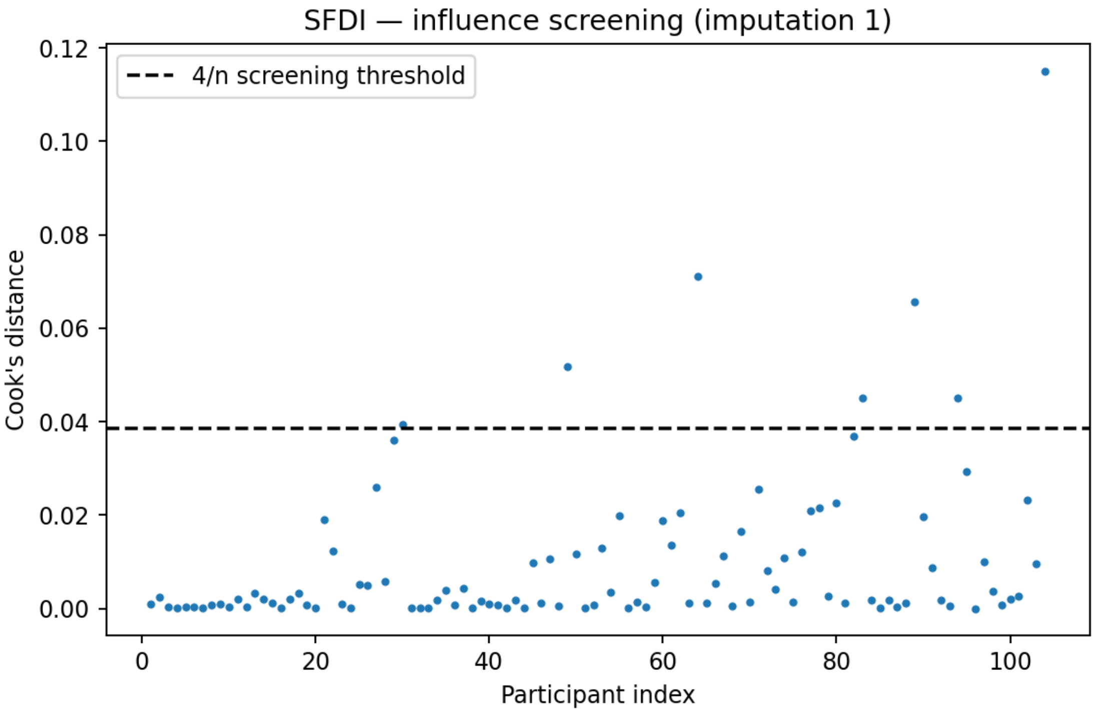

Figure S12 : six-week outcome regressed on randomized group and corresponding baseline outcome; representative completed dataset 1/50.

Complete-Case Analysis

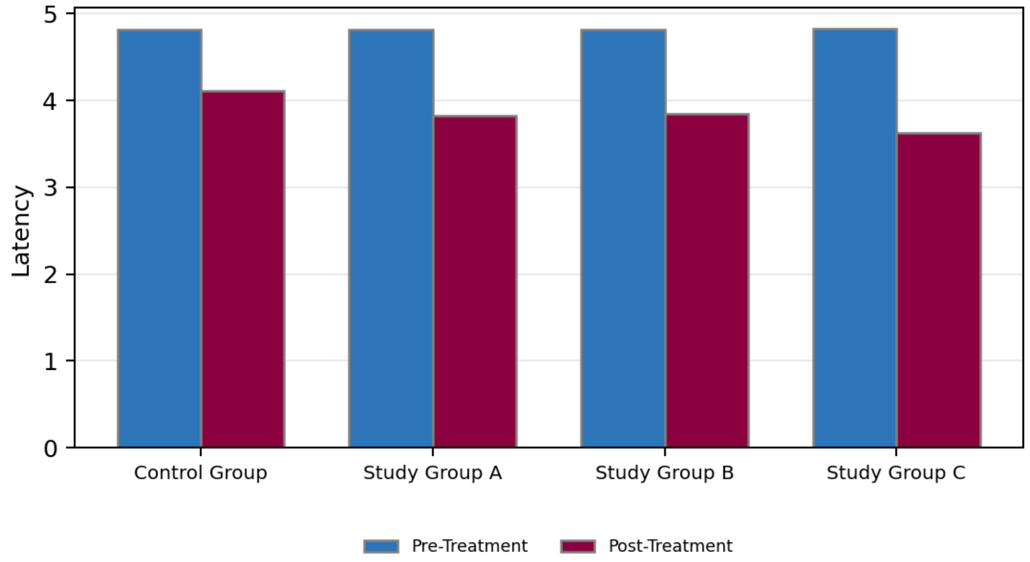

Figure S13 : **Mean latency values pre- and posttreatment in all groups**

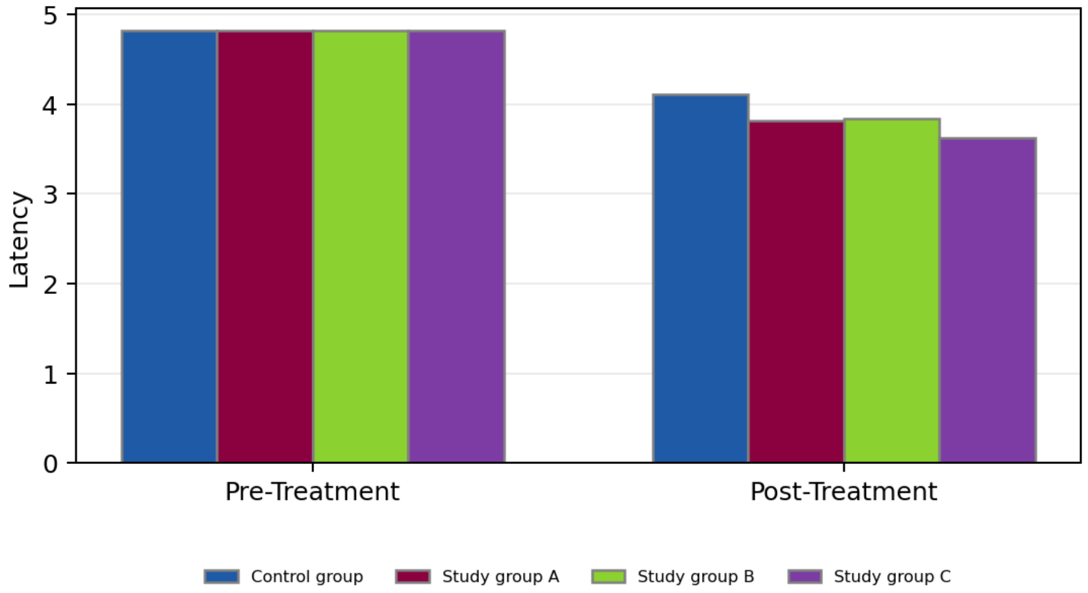

Figure S14 **: Mean latency values pre- and posttreatment among the groups.**

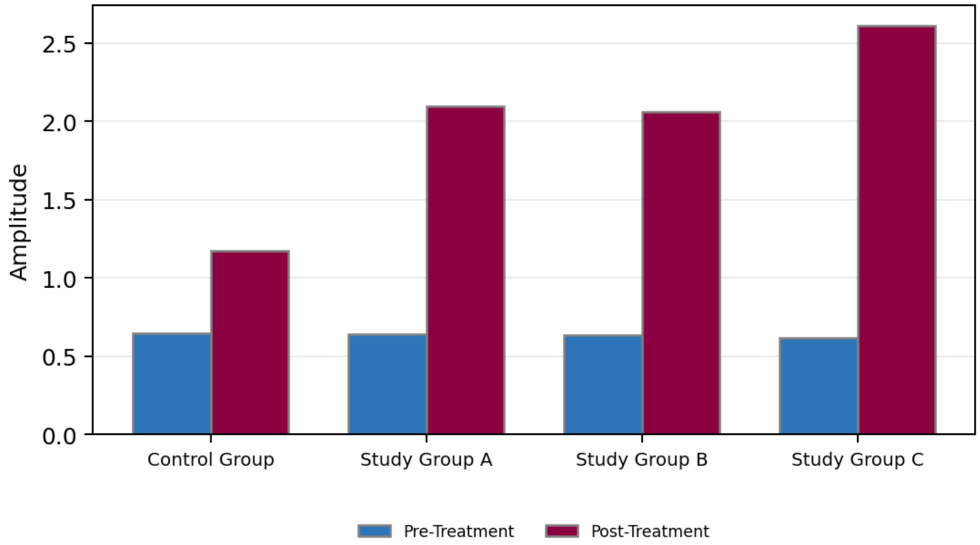

Figure S15 **: CMAP amplitude values pre- and posttreatment in all groups**

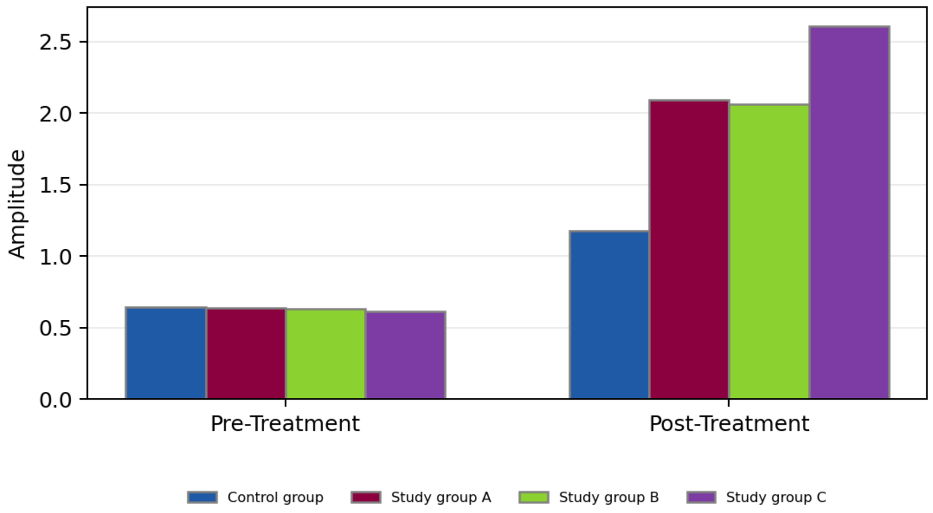

Figure S16 : **CMAP amplitude values pre- and posttreatment among the groups**

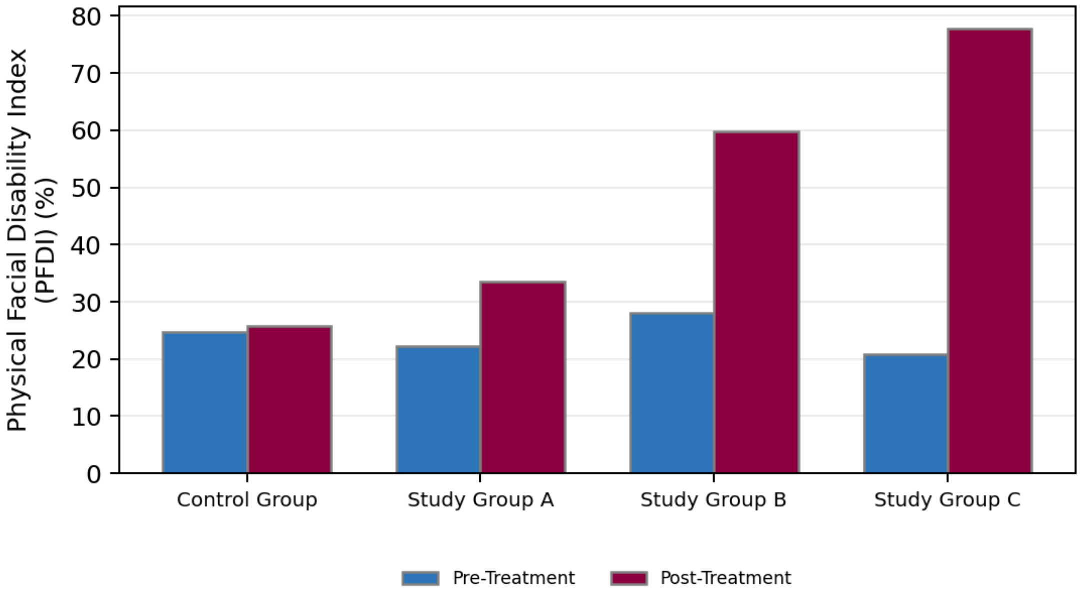

Figure S17 : **Mean PFDI values pre- and posttreatment in all groups**

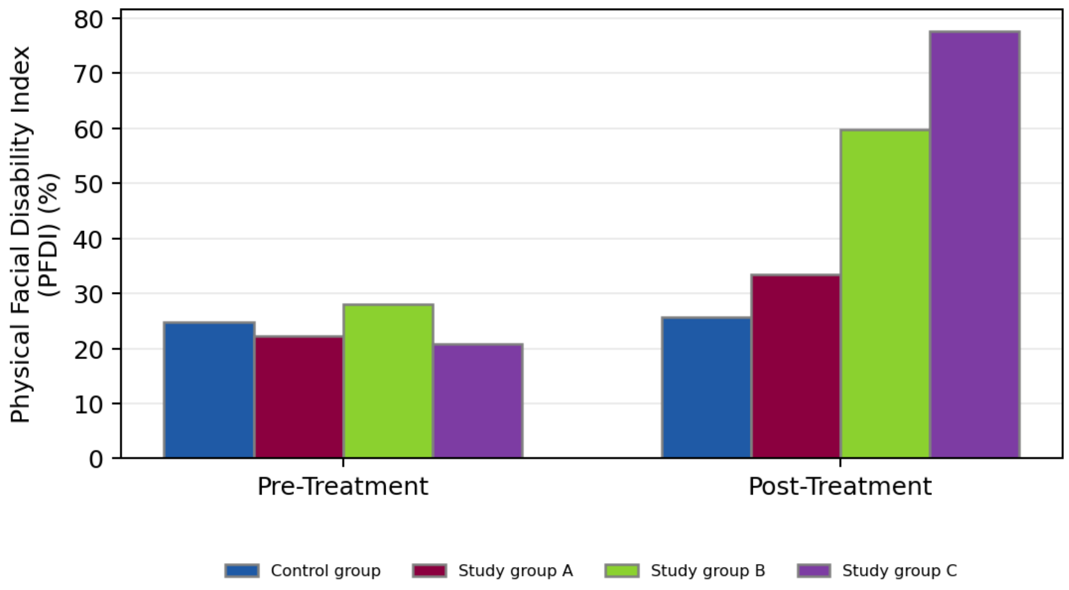

Figure S18 : **Mean PFDI values pre- and posttreatment among the groups**

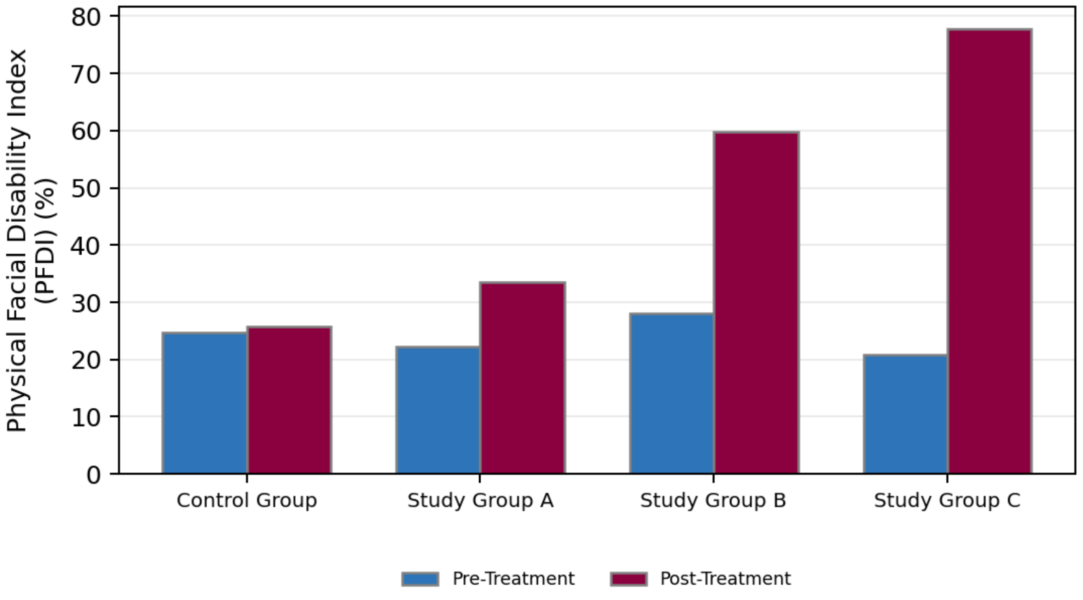

Figure S19 : **Mean SFDI values pre- and posttreatment in all groups**

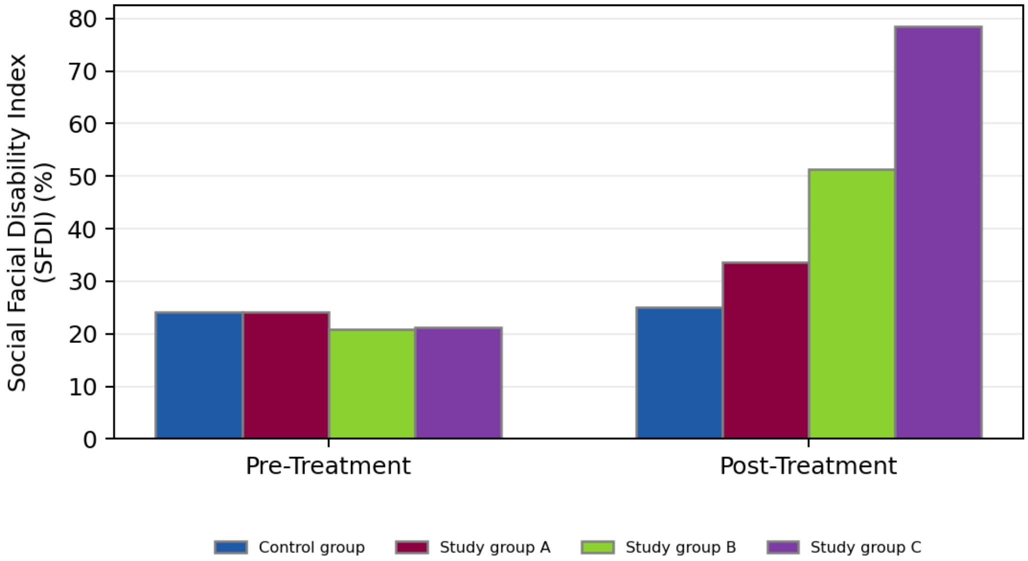

Figure S20 : **Mean SFDI values pre- and posttreatment among the groups**
